# Photodynamic Strategies for Prevention of Titanium Osteoimplant Infections

**DOI:** 10.34133/research.1092

**Published:** 2026-02-16

**Authors:** Junfeng Wang, Lian Guan, Weilin Sang, Libo Zhu, Guoqing Pan, Tao Wang, Jinzhong Ma

**Affiliations:** ^1^ Department of Orthopaedics, Shanghai General Hospital, Shanghai Jiao Tong University School of Medicine, Shanghai 201620, China.; ^2^ Department of Orthopedics, The Huai’an 82 Hospital, Huai’an, Jiangsu 223001, China.; ^3^ Institute for Advanced Materials, School of Materials Science and Engineering, Jiangsu University, Zhenjiang, Jiangsu 212013, China.

## Abstract

The growing demand for orthopedic implants, driven by an aging global population and the rising occurrence of bone abnormalities and defects, relies heavily on biocompatible titanium (Ti) and its alloys. However, their inherent biological inertness often results in poor osseointegration and increased vulnerability to bacterial infections, particularly those caused by biofilm-forming pathogens. Traditional antibiotic treatments frequently fail against biofilms and contribute to the escalating problem of antibiotic resistance, emphasizing the need for alternative therapies that do not foster resistance. Photodynamic therapy (PDT), which uses light-activated photosensitizers to generate reactive oxygen species (ROS) for effective bacterial eradication, presents a promising noninvasive strategy with high precision and low likelihood of resistance development. This review thoroughly explores the mechanisms of PDT and its application to Ti implants, focusing on both organic and inorganic photosensitizers. Moreover, considering current challenges, the study investigates approaches to improve PDT’s effectiveness in combating implant-related infections. These include optimizing PDT systems, developing new AIE-active photosensitizers, increasing oxygen supply to boost ROS production, combining multi-modal antibacterial treatments, and designing dual-functional implants. The review aims to identify current limitations and propose future directions, offering valuable insights to advance PDT-based strategies that enhance the antibacterial performance of Ti implants in the post-antibiotic era.

## Introduction

With the aging world population, the incidence of bone abnormalities due to different skeletal disorders, including traumatic injuries, bone cancers, and osteoarthritis, is rising, leading to an increased demand for orthopedic implants. Titanium (Ti) and its alloys are extensively utilized in orthopedic implants owing to their superior biocompatibility, exceptional mechanical strength, and corrosion resistance [1]. Nonetheless, their inherent biological inertness constrains their capacity to withstand bacterial invasion and facilitate bone integration, resulting in serious problems that considerably limit their clinical use [2]. Epidemiological research has indicated that about 19.3% of unsuccessful total knee arthroplasties in the United States are due to infection [3]. Some pathogenic bacteria can swiftly colonize and establish biofilms within hours of implantation. This microbial colonization competitively inhibits osteogenic cell adhesion, ultimately leading to inadequate bone integration [4,5]. Moreover, the development of dense biofilms renders conventional antibiotic therapies ineffective, while excessive antibiotic use has exacerbated the problem of bacterial resistance [6]. Extracting contaminated implants is frequently the sole viable solution, but this necessitates several procedures, which impose considerable health and financial strain on patients [7].

Due to the serious consequences of implant-related infections, healthcare systems have implemented extensive preventive measures, including standardized preoperative patient preparation, perioperative antibiotic prophylaxis with first- and second-generation cephalosporins, and strict surgical site disinfection [8]. Despite these precautions, the risk of infection after orthopedic implantation remains substantial, with a global incidence between 2% and 5% [9]. Bone implant-associated infections, especially those involving biofilm formation, present major treatment challenges. The protective barrier of microbial biofilms often renders traditional methods—such as systemic antibiotics combined with repeated surgical irrigation and debridement—ineffective at eliminating bacteria [10]. The absence of effective strategies to quickly break down established biofilms and prevent their recurrence highlights the urgent need to develop implant materials with inherent antibacterial properties, ensuring long-lasting therapeutic effects.

Recent studies have concentrated on 2 approaches to reduce infection risks associated with Ti-based implants: passive defense through anti-adhesive surface modifications and active offense via antibacterial surface engineering [11]. Given that the first bacterial attachment is a pivotal phase in biofilm development, passive methods to inhibit microbial adhesion by physicochemical surface alterations, including electrostatic repulsion and extreme wettability (superhydrophilicity or superhydrophobicity), are employed. Antifouling coatings made from poly(ethylene glycol) (PEG)-based polymers or zwitterionic substances have demonstrated excellent effectiveness in inhibiting bacterial colonization [12]. While superhydrophobic surfaces exhibit remarkable short-term antibacterial efficacy, their surface characteristics degrade over time, which may facilitate bacterial adhesion. Furthermore, their pronounced hydrophobicity can impede the adhesion and growth of eukaryotic cells such as osteoblasts, hence undermining osseointegration. In contrast, superhydrophilic coatings create a strong hydration barrier that physically obstructs bacterial access by diminishing interactions between germs and the surface. Nonetheless, the progressive deterioration of hydrophilic polymers may ultimately result in fouling and a reduction in long-term antibacterial efficacy [13].

To confer direct bactericidal properties to Ti-based implants, surfaces enhanced with antimicrobial agents, including antibiotics, antimicrobial peptides, enzymes, quaternary ammonium compounds (QACs), metallic or nonmetallic nanoparticles, and metal ions (e.g., Ag^+^, Zn^2+^, and Cu^2+^), have garnered considerable interest [10,14]. These functionalized surfaces exhibit extensive antibacterial efficacy against both Gram-positive and Gram-negative microorganisms. However, they encounter other constraints, such as the rapid exhaustion of active drugs, the potential emergence of microbial resistance from recurrent exposure, and cytotoxicity issues associated with ion leaching or nanoparticle release. Recent investigations have alarmingly indicated that bacteria may develop resistance to traditional antibiotics and inorganic agents such as silver nanoparticles after prolonged exposure [15]. Consequently, there is an urgent need to develop direct, noninvasive, and non-resistance-inducing alternative therapies to address the current challenges related to implant-associated infections in the post-antibiotic era.

Photodynamic therapy (PDT) is an innovative approach that employs photosensitizers, which produce reactive oxygen species (ROS) (e.g., ·OH, O₂·^−^, and O₂) upon activation by light of specific wavelengths, resulting in the disruption of cell membranes and the denaturation of biomolecules such as lipids, proteins, and nucleic acids, thereby effectively eliminating cancer cells or bacteria [16]. Owing to its noninvasive characteristics, spatiotemporal controllability, and low risk of resistance, PDT has been extensively utilized in anticancer and anti-infective treatments [17]. Numerous clinical studies have explored the potential of PDT as an alternative to conventional antibiotic-based treatments in combating infections. A meta-analysis with 5 randomized clinical studies (RCTs) included reveals that PDT presents similar clinical results compared to antibiotic therapy with amoxicillin plus metronidazole (AMX + MTZ) as adjuvants in the nonsurgical treatment of periodontitis [18]. Another research with 10 trials in periodontitis and 5 trials in peri-implantitis also supports PDT as equal clinical evidence as antibiotics in the treatment of periodontitis and peri-implantitis [19]. The investigations examining the impact of PDT on implant surface have found that PDT efficiently eliminates bacteria without impairing surface roughness [20]. Therefore, as a non-antibiotic and surgery-independent therapeutic method, PDT presents great promise for addressing implant-related infections in the dental and orthopedic fields.

While previous reviews have comprehensively detailed the mechanism of PDT, the photosensitizers utilized, and their applications in treating various cancers and microbial infections [16,17,21], PDT’s potential for managing deep-seated implant-associated infections remains considerably underexplored. Within the antimicrobial PDT domain, the existing literature predominantly focuses on superficial skin/wound infections and oral infections such as periodontitis and endodontics [22–24]. The application of PDT to combat infections surrounding deep-seated Ti implants, however, has received insufficient attention. Successfully implementing PDT on Ti implants presents a considerable challenge, given the inherent complexity of surface modification and the dual clinical imperatives of ensuring robust osseointegration while preventing infection [2]. Several critical issues demand clarification and resolution, including the selection of appropriate photosensitizers, optimization of irradiation wavelength and protocols, strategies for photosensitizer design and their immobilization onto Ti surfaces, and related biocompatibility and cytotoxicity assessments. To the best of our knowledge, a comprehensive review dedicated to this specific research domain remains absent. It is therefore imperative to conduct a timely synthesis that bridges this critical knowledge gap and paves the way for future investigations. This review provides a comprehensive elucidation of the fundamental processes and antibacterial mechanisms underpinning PDT, with a particular focus on the application of diverse photosensitizers to Ti implants. In light of the current limits, the latter part of the paper discusses and proposes several effective and actionable strategies for improvement, hoping to inspire future research endeavors and promote final clinical translation of PDT for combating Ti osteoimplant infections.

## Mechanisms of PDT

### Mechanisms of photodynamic reactions

Although the origins of PDT trace back to the early 1900s, its application in antimicrobial therapy emerged in the mid-1990s [25]. Successful PDT requires 3 essential components: photosensitizers, light of a suitable wavelength for excitation, and molecular oxygen in the surrounding environment. Photosensitizers generally demonstrate low toxicity to cells or tissues. Upon photon absorption from a suitable light source, these substances can interact with H₂O or O₂ via electron transfer or energy transfer to oxygen molecules, resulting in the production of ROS [16].

**Fig. 1.**
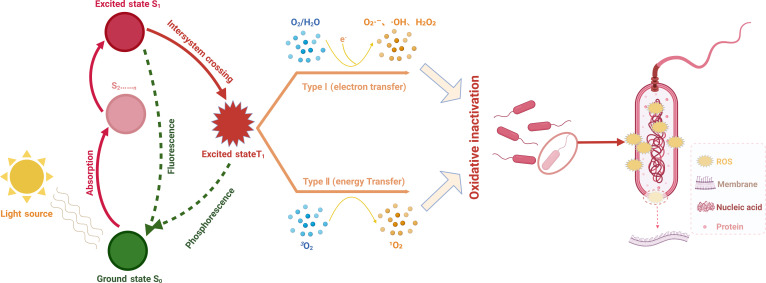
Schematic illustration of the mechanism of photodynamic therapy.

Two principal categories of photodynamic reactions exist, both of which have a comparable initial mechanistic process (Fig. 1). When exposed to light of a particular wavelength, the photosensitizer absorbs photons, prompting an electron to move from its ground state (S₀) to the singlet excited state (S₁), generating a singlet excited-state photosensitizer (S₁ photosensitizer). The S₁ photosensitizer possesses an exceedingly brief half-life (in the nanosecond range) and experiences 1 of 2 potential outcomes: (a) It reverts to the ground state (S₀) via nonradiative decay, dissipating the absorbed photon energy as heat, or (b) it undergoes intersystem crossing (ISC) to the more stable triplet excited state (T₁), which has a half-life in the microsecond range, thereby generating a triplet excited-state photosensitizer (T₁ photosensitizer) [16,17]. The T₁ photosensitizer is the active form capable of interacting with diverse substrates to produce ROS through 2 separate pathways.

Type I reactions entail electron transfer and hydrogen atom transfer, in which the T₁ photosensitizer directly engages in electron transfer or hydrogen abstraction with the substrate, resulting in the generation of free radical intermediates. These intermediates swiftly interact with biomolecules or oxygen, producing substantial quantities of ROS, including hydrogen peroxide (H₂O₂), superoxide anion (O₂·^−^), and hydroxyl radicals (·OH). The premise of type I reactions is that they are independent of oxygen levels, granting them good tolerance to hypoxic circumstances and making them uniformly effective against oxygen-deficient bacterial biofilm infections [26]. Conversely, type II reactions rely on energy transfer. The T₁ photosensitizer, with an identical spin to ground-state molecular oxygen, facilitates direct intermolecular energy transfer, resulting in the production of highly reactive singlet oxygen (^1^O₂). Given that the majority of organic compounds exist in the fundamental singlet state, except for oxygen molecules that possess a triplet ground state and can be excited to the singlet state, energy transfer occurs solely in the presence of O₂, rendering type II reactions oxygen-dependent [16]. Nonetheless, variables including bacterial metabolic activity, biofilm development, host immunological responses, and local acidification frequently result in a deficiency of O₂ in the bacterial infection microenvironment [27], substantially hindering the effectiveness of type II PDT. To solve the limitation of oxygen concentration during PDT, great efforts have been dedicated to developing targeted strategies toward oxygen delivery and production, which will be discussed in the latter part. In fact, type I and II reactions generally transpire together despite distinct mechanisms. The contribution ratio of both reactions is contingent upon various parameters, including local oxygen content, pH, photosensitizer characteristics, and tissue dielectric constant. As the ambient oxygen depletes, the type I mechanism becomes predominant [16].

Regardless of oxygen requirement, both type I and II PDTs offer prominent advantages over conventional antibiotic treatments, due to their light-activated ROS-dependent bactericidal nature [16,24]. The incapacity of bacteria to effectively detect oxidative stress and the impairment of cross-generational adaptability render PDT less susceptible to bacterial resistance, even following repeated treatments [28]. Unlike systemically administered antibiotics (oral/injectable), which lack tissue specificity, PDT enhances biosafety through spatial targeting of infected sites, thereby minimizing damage to healthy tissues. Furthermore, a principal advantage of PDT lies in its precise spatiotemporal control enabled by light activation, making it possible for customized therapeutic procedures via meticulous adjustment of irradiation settings and selection of appropriate photosensitizers. Consequently, these satisfactory characteristics, including broad-spectrum antimicrobial efficacy, markedly diminished bacterial resistance risks, high tissue selectivity with minimal side effects, and tailored treatment position PDT as a promising antibiotic alternative for infection control.

### Mechanisms of combating infections

#### ROS in PDT

As a potent arsenal, ROS plays a core role in PDT against bacterial infections. ROS attacks multiple bacterial targets, conferring PDT with broad-spectrum antibacterial capability and high eradication efficacy. Membrane disruption, intracellular oxidation, and genetic damage constitute the primary bactericidal mechanisms of ROS [29]. The oxidative impairment to membrane structures damages the cell membrane integrity, leading to the leakage of cellular contents and invasion of toxic substances into cells. Excess ROS triggers oxidative stress, inducing extensive oxidative destruction of intracellular components, such as various enzymes, structural proteins, and membrane receptors [30]. Oxidation of the critical metabolites like coenzyme A (CoA) can inhibit cell respiration, resulting in compromised adenosine triphosphate (ATP) synthesis and energy depletion [31]. Notably, ROS can break bacterial plasmid DNA and RNA molecules to cause cell death [32]. To gain a comprehensive knowledge of PDT’s antibacterial mechanism, it is essential to understand the fundamental physicochemical features of ROS, including type I (O₂·^−^, ·OH, and H₂O₂) and type II ROS (^1^O_2_).

·OH is a highly reactive and nonselective radical capable of rapidly oxidizing most organic compounds, making it among the most potent free radicals [33]. Due to its inability to traverse cell membranes, ·OH primarily inflicts damage at the surface, attacking structural components of cell membranes or walls. The reaction between ·OH and carbon–carbon double bonds in membrane lipids can generate substantial peroxides and hydroperoxides, which further disrupt bacterial membrane integrity [29]. Despite the shortest lifespan (about 3 × 10^−11^ s in aqueous solutions), ·OH possesses the highest cytotoxicity [26]. Another potent radical species is O₂·^−^, the one-electron reduction form of molecular oxygen. Due to defensive superoxide dismutase (SOD) catalyzing O₂·^−^ dismutation to H₂O₂, the intracellular lifespan of O₂·^−^ is very brief (<50 ms), limiting its diffusion distance to approximately 40 μm [34]. In biological systems, the pH-dependent protonation also converts O₂·^−^ to hydroperoxyl radicals (HOO·) [33]. While negatively charged O₂·^−^ is repelled by anionic membrane phosphates, HOO· can penetrate the cell membrane and accumulate in hydrophobic membrane domains, collaborating with other radicals to initiate lipid peroxidation chain reactions and inactivate radical-scavenging enzymes, leading to catastrophic membrane damage and cell dysfunction [35].

H₂O₂, with extended lifespan (1 to 3 min in mammalian cells) and thermodynamic stability, provides sustained antibacterial activity. Due to its high p*K*_a_ (where *K*_a_ is the acid dissociation constant) value, H₂O₂ can easily diffuse between cells and cross cell membranes to disrupt intracellular proteins/enzymes, particularly those with -SH groups [26]. Under physiological conditions, its decomposition is mostly via enzymatic reaction mediated by peroxidase (POD) and catalase (CAT). However, when in oxidative stress, POD catalyzes the decomposition of H₂O₂ to highly toxic ·OH, thereby exacerbating damage to cellular components [26]. Notably, H₂O₂ can be enzymatically broken down by CAT to generate oxygen, which offers a promising solution to the hypoxia limitation for enhanced PDT [26,34]. For example, Huang et al. [36] leveraged the cascade reaction of glucose oxidase and CAT to convert the excessive glucose in diabetic abscess tissue into oxygen, which markedly alleviated the hypoxia in the infected region, ensuring sufficient photogenerated ROS to combat pathogenic methicillin-resistant *Staphylococcus aureus* (MRSA).

Although H₂O₂ is a strong oxidizer with a standard redox potential of 1.77 V, it exhibits minimal or no interaction with the majority of biomolecules. The cytotoxicity of H₂O₂ largely hinges on its capacity to transform into ·OH [26]. Haber–Weiss reaction is a well-known process that can integrate H₂O₂ with O₂·^−^ to generate ·OH in the presence of transition metals. Besides, H₂O₂ can also react with HOO· for ·OH generation. When reducing metal ions (e.g., Fe^2+^ and Cu^+^) exist, H₂O₂ undergoes Fenton or Fenton-like reactions through single-electron reduction, yielding ·OH with considerably higher efficiency than the Haber–Weiss pathway [34]. The Fenton reaction constitutes the fundamental basis for chemodynamic therapy (CDT) [37], which has motivated the integration of PDT with CDT. In the work of Tian et al. [38], MIL-101 (Fe)-NH_2_, TA, and Ce6 were integrated into a unified platform to produce a novel pH-responsive nanophotosensitizer (MT@Ce6). Under acidic conditions, MT@Ce6 shifted ROS dominance from photogenerated short-lived ^1^O_2_ to highly penetrative ·OH through Fenton reaction driven by the released Fe^2+^. The superior oxidative capacity of ·OH effectively boosted the antibiofilm efficacy of PDT. Li et al. [39] developed a biofilm microenvironment-responsive nanoplatform consisting of copper-doped bovine serum albumin (BSA) loaded with CuO_2_ and indocyanine green (ICG), which was capable of releasing Cu^2+^ and H₂O₂ in a slightly acidic environment. Under 808-nm laser irradiation, the loaded photosensitizer ICG not only triggered the generation of ^1^O₂ but also provided photonic hyperpyrexia that further promoted the Fenton-like reaction of Cu^2+^ for enhanced ·OH production while inducing thermal decomposition of CuO_2_ for O_2_ supply. This nanoplatform, augmenting PDT and CDT via O_2_/H_2_O_2_ self-supplying, showed great potential in treating biofilm infections.

The above 3 are efficient antibacterial executors of type I PDT, while ^1^O_2_ serves as a principal bactericidal agent in type II PDT. ^1^O₂ is the excited state of molecular oxygen, with a vacant orbital in its outermost electron shell [40]. Due to its unique property, ^1^O₂ readily reacts with unsaturated organic compounds via electrophilic addition and electron extraction. For instance, ^1^O₂ attacks unsaturated fatty acids in cell membranes through Alder-ene reactions, inducing hydroperoxide formation that ultimately triggers lipid peroxidation. Electron transfer between ^1^O₂ and adjacent lipids or thiols also occurs in certain reactions to generate free radicals [40]. Amino acid residues such as methionine, histidine, and tryptophan are particularly susceptible to ^1^O₂ oxidation, resulting in structural alterations and functional impairment of proteins [41]. Furthermore, ^1^O₂ induces the oxidation of guanine, thereby causing irreversible oxidative damage to DNA [42]. The active interactions between ^1^O₂ and cellular components constrain its lifespan to just 10 to 40 ns and diffusion range of only 10 to 20 nm [41].

Indeed, ROS is constitutively generated during normal cellular metabolism under physiological conditions. The inherent antioxidant defense system of bacteria, especially enzymatic ROS scavengers represented by SOD, CAT, POD, and glutathione reductases, constantly eliminates cellular ROS and mitigates ROS-induced oxidative stress, hence preserving intracellular redox equilibrium [43]. However, when exposed to PDT, sustained overproduction of ROS that overwhelms cellular clearance capacity will disrupt this delicate redox homeostasis, resulting in substantial ROS accumulation, which triggers a lethal cascade of irreversible biomolecular and structural damage. While cancer cell resistance to PDT has been documented and concerns persist about ROS resistance developing analogously to antibiotic resistance [44], bacterial resistance to PDT-induced ROS bursts has yet to be reported.

#### PDT-activated immune responses

While the direct bactericidal effect of ROS is widely recognized as the primary mechanism in PDT, the immunoinductive effect of PDT also plays a non-negligible role. PDT can elicit acute inflammatory responses at the action site, resulting in the release of numerous proinflammatory mediators, including tumor necrosis factor-α (TNF-α), interleukin-6 (IL-6), IL-1, complement proteins, heat shock proteins (HSPs), and arachidonic acid metabolites, alongside the up-regulation of adhesion molecules such as E-selectin and intracellular adhesion molecule-1 (ICAM-1). These molecular events collectively facilitate the infiltration and activation of immune cells to initiate both innate and adaptive immune responses and are considered crucial for the activation of antitumor immunity [25,45]. Despite being a localized treatment, PDT’s immunostimulatory effects may extend beyond the primary site. PDT can induce a robust acute-phase response, characterized by elevated levels of serum C-reactive protein (CRP), mannose-binding lectins (MBLs), and serum amyloid P component (SAP), thereby augmenting the overall immune responses against tumors [46]. Furthermore, the local and systemic inflammatory responses initiated by PDT can reciprocally augment the adaptive immune response, thereby protecting the host organism in an antigen-specific manner [45]. Research has highlighted the pivotal role of dendritic cells in PDT-enhanced adaptive antitumor immunity. By recognizing damage-associated molecular patterns (DAMPs)/cell death-associated molecular patterns (CDAMPs) released or exposed by dying tumor cells, dendritic cells undergo activation and transformation into functional antigen-presenting cells (APCs), thereby effectively priming CD4^+^ T helper cells and CD8^+^ cytotoxic T lymphocytes to initiate adaptive immunity [45,47].

Macrophages and neutrophils are crucial to the host’s innate immune defenses [46]. The impact of PDT on macrophages appears to be double-edged. In the process of PDT, macrophages are found to secrete IL-6, TNF-α, and IL-10. While the former 2 are classic proinflammatory cytokines, IL-10 can impede phagosome maturation and suppress proinflammatory cytokine production. This phenomenon is presumably associated with the therapeutic dosage [48]. Other studies confirm that PDT enhances macrophage phagocytic activity and facilitates intracellular pathogen clearance [49]. The great role of innate immunity in PDT is further corroborated by an in vivo investigation conducted by Tanaka et al*.* [50]. In their research, following administration of antibodies targeting GR-1 (neutrophil marker) and proinflammatory mediators, the therapeutic effects of PDT underwent a drastic reduction in the murine model of MRSA arthritis. This result indicates that, beyond bactericidal effects induced by ROS, neutrophil accumulation and phagocytic activity at infection sites are essential mechanisms for achieving PDT-mediated bacterial removal. Collectively, these studies underscore the indispensable role of host immune defenses in PDT-mediated antibacterial therapy, albeit the precise mechanisms and regulatory networks still require further investigations and clarification.

Biofilm infection poses a major challenge to clinical implants. Following the colonization of biomedical interfaces, bacteria continuously secrete extracellular polymeric substances (EPSs), including exopolysaccharides, extracellular DNA, proteins, and lipids, to facilitate surface adhesion and aggregation [5]. When the bacterial population density reaches a certain threshold, the quorum-sensing system is activated and regulates the expression of various genes associated with biofilm formation, ultimately leading to the development of architecturally sophisticated 3-dimensional (3D) biofilms [51]. Functioning as a physical barrier, these biofilms impede contact between APCs and pathogens while altering pathogen antigenicity or concealing critical immunogenic epitopes, thereby hindering sustained and effective recognition of pathogen-specific antigens by APCs. Furthermore, EPS severely obstructs the transportation of nutrients and oxygen into the biofilm. The hypoxia state, nutrient deprivation, and mildly acidic microenvironment surrounding implants create an immunosuppressive milieu, where the exhaustion of APCs, insufficient T cell activation, weakened antibody responses, and the generation of regulatory T cells (T_regs_) related to immune suppression and tolerance collectively lead to ineffective immune responses that permit bacterial persistence [51,52]. Therefore, it is important to reshape the immunosuppressive microenvironment within the biofilm for bacterial eradication.

Inspired by the immunomodulatory potential of PDT, Jiang et al. [53] developed vancomycin-grafted and Ce6-loaded BSA-MnO₂ nanoclusters (BMCV NPs) to disrupt biofilm barriers and reverse biofilm-induced immunosuppressive microenvironments. Owing to vancomycin’s targeting capability, BMCV NPs could specifically accumulate at the *S. aureus* infection site. Upon laser irradiation, photogenerated ROS efficiently destroyed extracellular DNA in the biofilm, undermining its barrier integrity. Concurrently, ROS-induced bacterial lysis triggered bacteria-like immunogenic cell death (ICD), accompanied by the release of abundant costimulatory factors. These factors, enhanced by immunoadjuvant manganese ions, managed to activate macrophage polarization and dendritic cell maturation, thereby enhancing antigen-presenting capability and amplifying adaptive immune responses. This photo-immunomodulatory strategy successfully eliminated biofilm-associated infections and established enduring immunological memory to avert recurrence. To address the high recurrence risk of postoperative MRSA infections, Tang et al. [54] developed ultra-micro AgB nanodots (NDs) serving as dual-functional antimicrobial photosensitizers and immunoenhancers. By combining photothermal and photodynamic therapeutic modalities, the immunostimulant-like AgB NDs leveraged exogenous stimuli, such as abrupt temperature spikes and excessive ROS generation, to induce immunogenicity and evoke a robust ICD response. As a result, AgB ND treatment effectively enhanced dendritic cell maturation, macrophage polarization, and T cell activation against the existing infection while also fostering long-term B cell-mediated immunological memory against re-invading MRSA. Consequently, harnessing and amplifying the immunoinductive effect of PDT warrants concentrated attention in forthcoming antibiofilm research.

In summary, PDT utilizes light-activated photosensitizers to generate ROS via type I and II photochemical processes. The potent therapeutic efficacy primarily stems from dual synergistic mechanisms: direct ROS-mediated bacterial inactivation and activation of host immune responses. Although the latter mechanism remains to be validated and completely elucidated, the integration of PDT with augmented immunotherapy is a highly promising therapeutic strategy for preventing biofilm infection occurrence and reoccurrence.

## PDT for Ti Implants

**Fig. 2.**
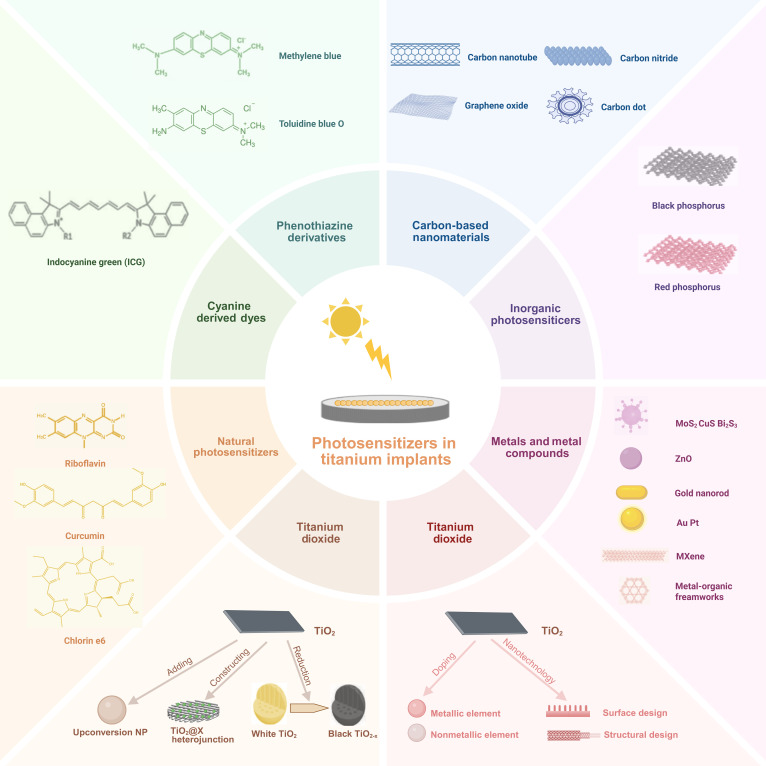
Schematic diagram of photosensitizers utilized in titanium implants.

Ti implants are frequently employed in orthopedics and dentistry. Nonetheless, unmodified Ti implants lack inherent antimicrobial characteristics. Diverse surface modification techniques, such as the insertion of antibacterial agents, integration of metallic or nonmetallic materials, and grafting of bioactive compounds, have been investigated and applied to meet the therapeutic demand for treating microbial infections [10]. The integration of Ti implants with photosensitizers has emerged as a promising solution for implant-associated infections (Fig. 2). Since the first utilization of eosin as the pioneering photosensitizer, these agents have evolved through 3 distinct generations of development: The first generation comprises porphyrin derivatives; the second generation mainly encompasses porphyrins, phthalocyanines, fused quinones, and metal phthalocyanines; and the third generation integrates second-generation photosensitizers with carrier biomolecules or targeting agents to reduce off-target effects and optimize pharmacokinetics [55]. For orthopedic and dental implant applications, photosensitizers are broadly classified into 2 categories: organic molecular agents and inorganic functional materials (as listed in Table 1).

**Table 1.** Summary of the reported photosensitizers in photodynamic therapy to prevent titanium implant infections

Photosensitizers	Incorporation methods and materials	Light settings	Antimicrobial mechanism	Antimicrobial efficacy	Ref
Curcumin (Cur)	Physical coating Ti-PDA-Cur	405 + 808 nm (10 min)	Type II PDT + PTT	Both 100% against *E. coli* and *S. aureus* in vitro	[70]
Flavin mononucleotide (FMN)	Physical coating Ti-FMN	Blue LED (3.7–4 W cm^−2^ for 10 s)	Type I/II PDT	About 93% against *S. aureus* in vitro	[76]
Chlorin e6 (Ce6)	Physical coating Ti/GelMAc/MPDA@Ce6	660 nm LED (1 W cm^−2^ for 10 min)	Type II PDT	88.55% against *E. coli* and 85.60% against *S. aureus* in vitro	[79]
Methylene blue (MB)	Physical coating Ti-MB	664 nm LED (15 J cm^−2^ for 10 min)	Type I/II PDT	MBC_100%_: 0.5–5 μg/ml for planktonic bacteria and 50–100 μg/ml for biofilms	[60]
Methylene blue (MB)/toluidine blue (TBO)	Physical coating Ti-MB/Ti-TBO	670 nm LED (160 mW for 60 s)	Type I/II PDT	85.5% of MB and 96.6% of TBO against *S. aureus* in vitro	[62]
Indocyanine green (ICG)	Physical coating Ti-PSACT/CNPs-ICG	810 nm LED (31.2 J cm^−2^ 1 min) + 1 MHz US (1.56 W cm^−2^ for 1 min)	Type II PDT + SDT	90.5% against polymicrobial periopathogenic biofilms in vitro	[65]
Indocyanine green (ICG)	Physical coating Ti-MPDA/ICG/RGD	808 nm NIR (1 W cm^−2^ for 30 min in vitro and 2.0 W cm^−2^ for 10 min in vivo)	Type II PDT + PTT	99.06% against *S. aureus* biofilms in vitro and 95.4% in vivo	[66]
New indocyanine green (IR820)	Physical coating LA-NICG-PDA (LIP)@SrTiO3	808 nm NIR (1.04 W cm^−2^ for 20 min)	Type II PDT + PTT + NO	Over 96% against *S. aureus* in vivo	[192]
IR780	Physical coating Ti-RP-IR780-RGDC	808 nm NIR (0.5 W cm^−2^ for 10 min in vitro and 2.0 W cm^−2^ for 10 min in vivo)	Type II PDT + PTT	89.3% against *S. aureus* in vitro and 96.2% in vivo	[69]
IR780	Chemical grafting Ti-Lip(IR780 + PFH)	808 nm NIR (1 W cm^−2^ for 15 min)	Type II PDT	99.62% against *E. coli* and 99.63% against *S. aureus* in vitro	[181]
AIEgen (4BC)	Precipitation adsorption method 4BC@TMP scaffold	365 nm UV (250 mW/cm^2^ for 20 min)	Type II PDT	98.1% against *S. aureus* in vitro	[174]
Titanium dioxide (TiO_2_)	Hydrothermal method TiO_2_:FYH/Cur/BMP-2	1,060 nm NIR-II (0.6 W cm^−2^ for 15 min, both in vitro and in vivo)	Type I/II PDT + PTT + Quorum-sensing inhibition + Physical puncture	95.2% against *S. aureus* in vitro and 92.3% in vivo	[86]
Titanium dioxide (TiO_2_)	Hydrothermal method PDA/p-TiCu-300 °C	808 nm NIR (1.5 W cm^−2^ for 15 min) + US (1.5 W cm^−2^ for 5 min)	Type I/II PDT + PTT + SDT	99.19% against *E. coli* and 95.03% against *S. aureus* in vitro	[89]
Titanium dioxide (TiO_2_)	Hydrothermal method Titanate/TiO_2−*x*_ heterostructure	808 nm NIR (0.7 W cm^−2^ for 15 min in vitro and 1 W cm^−2^ for 10 min in vivo)	Type I/II PDT + PTT	98.33% against *E. coli* and 98.78% against *S. aureus* in vitro; 94.45% against *S. aureus* in vivo	[90]
Titanium dioxide (TiO_2_)	Successive ionic layer adsorption and reaction PDA/CuS/TiO_2_ heterojunction	808 nm NIR (0.7 W cm^−2^ for 7 min in vitro and in vivo)	Type I PDT + PTT	Both 99% against *E. coli* and *S. aureus* in vitro; 99.06% against *S. aureus* in vivo	[93]
Titanium dioxide (TiO_2_)	Hydrothermal method TiO_2_/Bi_2_WO_6_ piezoelectric heterojunction	808 nm NIR (1 W cm^−2^ for 10 min in vitro and in vivo)	Type I/II PDT + PTT	99.03%, 99.11%, and 98.31% against *E. coli*, MRSA, and *P. gingivalis*, respectively, in vitro; 97.81% against MRSA in vivo	[205]
Titanium dioxide (TiO_2_)	Hydrothermal method TiO_2_ nanorod	808 nm NIR (0.8 W cm^−2^ for 15 min)	Type I/II PDT + PTT + Physical puncture	Both over 99% against *E. coli* and *S. aureus* in vitro	[102]
Titanium dioxide (TiO_2_)	Alkaline-acid bidirectional hydrothermal method TiO_2_/TiO_2−*x*_ metasurface	808 nm NIR (0.5 W cm^−2^ for 10 min in vitro and 1.4 W cm^−2^ for 10 min in vivo)	Type I/II PDT	96.88% against *E. coli* and 97.56% against *S. aureus* in vitro; 91.8% against *S. aureus* in vivo	[106]
Titanium dioxide (TiO_2_)	Alkaline-acid bidirectional hydrothermal method TiO_2_ metasurface	808 nm NIR (1.1 W cm^−2^ for 10 min)	Type I/II PDT	99.27% against *E. coli* and 96.87% against *S. aureus* in vitro	[107]
Titanium dioxide (TiO_2_)	Hydrothermal + plasma-enhanced chemical vapor deposition (PECVD) TiO_2_-G metastructure/DOX	808 nm NIR (1.1 W cm^−2^ for 5 min in vitro and in vivo)	Type I/II PDT + PTT + DOX	97.36% against *E. coli* and 96.42% against *S. aureus* in vitro; 99.48%	[200]
Titanium dioxide (TiO_2_)	Chiral TiO_2_ superparticles	808 nm circularly polarized light (CPL) (0.2 W cm^−2^ for 10 min)	Type I/II PDT	100% against *E. coli* and 99.4% against *S. aureus* in vitro	[108]
Black phosphorus (BP)	Physical coating GD@pBP/PPENK	808 nm NIR (1 W cm^−2^ for 10 min)	Type II PDT + PTT	85% against *E. coli* and over 70% against *S. aureus* in vitro	[155]
Red phosphorus (RP)	Physical coating Ti-calcium titanate (CTO)/RP	808 nm NIR (0.5 W cm^−2^ for 20 min in vitro and 0.8 W cm^−2^ for 20 min in vivo)	Type I/II PDT + PTT	99.61% against *E. coli* and 99.78% against MRSA in vitro; 99.42% against MRSA in vivo	[157]
Red phosphorus (RP)	Physical coating Ti-RP/PCP/RSNO	808 nm NIR (1 W cm^−2^ for 20 min, both in vitro and in vivo)	Type I PDT + ·ONOO^−^ + PTT	Over 93.1% against MRSA in vitro and 99.2% in vivo	[194]
Carbon dots (CDs)	CDs-doped TiO_2_ nanorod	660 + 808 nm (both 0.6 W cm^−2^ for 15 min in vitro and in vivo)	Type I/II PDT + PTT	99.9% against *S. aureus* in vitro and 85% in vivo	[111]
Carbon quantum dots (CQDs)	Physical coating AgBiS2@CQDs/Ti	1,064 nm NIR-II (1 W cm^−2^ for 10 min in vitro and in vivo)	Type I/II PDT + PTT	91.1% against *E. coli* and 92.7% against *S. aureus* in vitro; 99.3% against *S. aureus* in vivo	[112]
Graphene oxide (GO)	Physical coating Ti/PDA/Ag_3_PO_4_/GO	660 nm VL (170 mW for 15 min)	Type I PDT + Ag^+^	99.53% against *E. coli* and 99.66% against *S. aureus* in vitro	[118]
Graphene oxide (GO)	Physical coating GO/NCD/Hap/Ti film	808 nm NIR (0.5 W cm^−2^ for 15 min in vitro and in vivo)	Type I PDT + PTT + extracellular electron transfer	99.8% against *E. coli* and 98.9% against *S. aureus* in vitro; over 98.1% against *S. aureus* in vivo	[119]
Carbon nitride (C_3_N_4_)	Physical coating MnO_2_/g-C_3_N_4_-Ti	VL (200 mW/cm^2^ for 20 min)	Type I/II PDT + MnO_2_ oxidization	99.26% against *E. coli* and 99.96% against *S. aureus* in vitro	[122]
ZnO	Physical coating Ti/CNTs/CS-ZnO	UV (30 min)	Type I PDT + Zn^2+^	Over 73% against *E. coli* and 98% against *S. aureus* in vitro	[115]
MoS_2_	Physical coating Chitosan@MoS_2_-Ti	660 + 808 nm (both 0.5 W cm^−2^ for 10 min)	Type II PDT + PTT	99.84% against *E. coli* and 99.65% against *S. aureus* in vitro	[129]
MoS_2_	Physical coating Chitosan /Ag/MoS_2_-Ti	660 nm VL (0.898 W cm^−2^ for 10 min)	Type I PDT + PTT + Chitosan	99.77% against *E. coli* and 98.66% against *S. aureus* in vitro	[130]
CuS	Physical coating Ti-PEG-Cu_2−*x*_S	808 nm NIR (1 W cm^−2^ for 5 min)	Type II PDT + PTT + Antifouling	99.96% against *E. coli* and 99.66% against *S. aureus* in vitro	[11]
Bi_2_S_3_	Physical coating Bi_2_S_3_ @Ag_3_PO_4_ /Ti	808 nm NIR (0.5 W cm^−2^ for 3 min and 0.25 W cm^−2^ for 12 min in vitro; 1 W cm^−2^ for 15 min in vivo)	Type I PDT + PTT + Ag^+^	99.74% against *E. coli* and 99.45% against *S. aureus* in vitro; over 94.54% against *S. aureus* biofilm in vivo	[134]
Au nanoparticles	Physical coating Au-RE/TiO_2_	980 nm NIR (625 mW/cm^2^ for 15 min)	Type I PDT + PTT + extracellular electron transfer	97.67% against *S. aureus* in vivo	[4]
Ag–Cu nanoparticles	Electrochemical deposition Ti/PPy/HA/Cu/Ag	808 nm NIR (1 W cm^−2^ for 10 min in vitro)	Type I/II PDT + PTT + Ag^+^/Cu^2+^	100% against *E. coli* and *S. aureus* in vitro	[146]
MXene/CaO_2_ heterojunction	Physical coating MXene/CaO2/SPEEK	808 nm NIR (1 W cm^−2^ for 15 min in vitro and in vivo)	Type I/II PDT + PTT + Ca^2+^-induced competent cell-like stage	Nearly 100% against *S. aureus* and MRSA in vitro; 98.7% against *S. aureus* in vivo	[137]
Ni(OH)_2_@CaTiO_3_ heterostructure	Physical coating Ni(OH)_2_@CaTiO_3_/Ti	808 nm NIR (0.55 W cm^−2^ for 15 min)	Type I PDT	95.5% against *E. coli* and 93.8% against *S. aureus* in vitro	[202]

### Organic photosensitizers

#### Phenothiazine derivatives

**Fig. 3.**
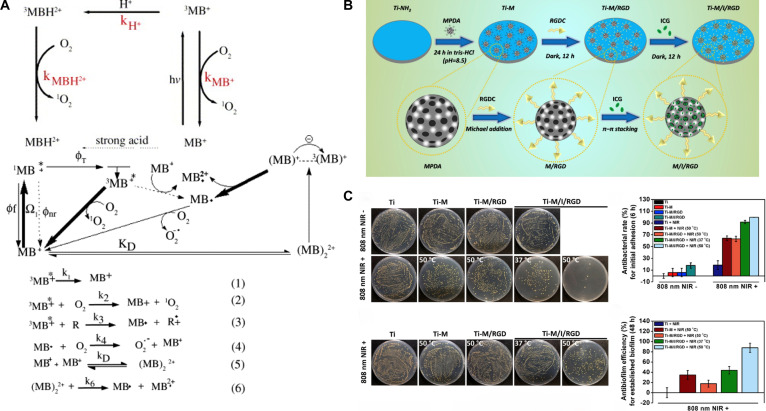
(A) Scheme of methylene blue photochemical reaction routes where MB, ^+1^MB, and ^+3^MB are methylene blue ground state, singlet, and triplet excited states, respectively, Ω_1_ is light absorption, ϕ_f_, ϕ_nr_, and ϕ_T_ are fluorescence, nonradiative, and triplet quantum yield, respectively. Reproduced with permission [58]. Copyright 2005, Elsevier. (B) Diagram of the fabrication route of Ti-M/I/RGD substrates. (C) Antibacterial and antibiofilm properties of Ti-M/I/RGD in vitro. Reproduced with permission [66]. Copyright 2019, Elsevier. (For interpretation of the references to color in this figure legend, the reader is referred to the web version of this article.)

Phenothiazine-based photosensitizers are the most commonly used in therapeutic applications. Methylene blue (MB) and toluidine blue O (TBO) are notable examples that have received approval from the U.S. Food and Drug Administration (FDA) and been widely utilized in dental implants for the treatment of periodontitis [56]. As a low-molecular-weight, cationic, and hydrophilic photosensitizer, MB can readily traverse the cell membranes of Gram-negative bacteria through porin channels and engage with lipopolysaccharides (LPSs) [57]. MB absorbs light in the red spectrum, exhibiting pronounced absorption between 600 and 660 nm. Environmental pH and concentration considerably influence the photodynamic behavior of MB. At low concentrations, MB typically transitions from the ground state to the triplet state upon light exposure, thereafter decaying to the ground state while transferring energy to oxygen molecules, producing ^1^O₂ via type II processes. As the concentration of MB increases, MB molecules spontaneously dimerize. In the excited state, these dimers form semi-reduced or semi-oxidized free radicals by electron transfer, which subsequently react with oxygen to produce O₂·^−^ [58]. Consequently, the elevation in MB concentration can facilitate a transition from type II PDT to type I PDT (Fig. 3A). Furthermore, it has been documented that ambient pH affects the rate of MB-sensitized photodegradation and dictates the yield of ^1^O₂ [58].

The application of MB-based PDT on the disinfection of dental and orthopedic implants has received a lot of attention. Huang et al. [59] reported that antimicrobial PDT with higher pH (pH 10) and higher MB concentration (200 μg/ml) could greatly diminish the adherence of *Aggregatibacter actinomycetemcomitans* and *Porphyromonas gingivalis* on Ti surfaces, as well as the LPS concentration. Moreover, an in vitro investigation revealed that MB-PDT effectively eradicated common bacteria associated with prosthetic joint infection (PJI) on orthopedic materials such as polyethylene, Ti alloys, cobalt–chromium alloys, and bone cement, without degrading the materials [60]. The identified minimum bactericidal concentration exhibiting 100% killing (MBC_100%_) was 0.5 to 5 μg/ml for planktonic bacteria and 50 to 100 μg/ml for biofilms, both of which fall within the established safety threshold of 10 mg/ml, thereby establishing MB-based PDT as a promising approach to prevent PJI and obviate the revision of medical prostheses.

TBO is likewise a tiny, hydrophilic, cationic photosensitizer that can be stimulated by red light in the 600- to 660-nm region. TBO’s minimal excitation energy, elevated membrane permeability, and capacity to form dimers render it an optimal option for binding to microbial cell membranes. In comparison to MB, TBO exhibits a more potent interaction with LPS, demonstrating superior bactericidal efficacy against Gram-negative bacteria [61]. Park et al. [62] discovered that PDT in conjunction with TBO and MB markedly diminished the development of *S. aureus* biofilm on sandblast and acid etching (SLA)-treated Ti surfaces. PDT utilizing TBO as a photosensitizer was more efficacious than MB alone, which may be attributed to TBO’s higher solubility and concentration in the bacterial cells.

#### Cyanine-derived dyes

Cyanine dyes, consisting of 2-terminal heterocyclic units connected by a polyacetylene bridge core structure, are among the most prevalent photosensitizers. ICG, characterized as an anionic near-infrared (NIR)-absorbing fluorescent contrast agent, is extensively utilized in medical imaging and PDT for diverse tumors [63]. As the sole cyanine dye sanctioned by the U.S. FDA, ICG exhibits optimal absorption in the NIR light spectrum (800 to 810 nm). The exceptional fluorescent properties, minimal dark toxicity, specific targeting capability, and high ^1^O₂ generating capacity set it apart from several homogeneous alternatives. Nonetheless, the brief half-life, inadequate stability, and vulnerability to biodegradation may restrict its effectiveness in PDT [64]. Moreover, in contrast to the photodynamic effect, the photothermal effect is more dominant, turning the majority of absorbed NIR light energy (about 88%) into thermal energy [63]. To augment the efficacy of PDT, ICG is frequently amalgamated with additional agents. Chitosan, a natural polysaccharide, is widely utilized as a biomaterial carrier for drug delivery owing to its distinctive properties. The positively charged chitosan can interact with negatively charged ICG to form complexes via ionic or hydrogen bonding and hydrophobic interactions. Chitosan nanoparticle-encapsulated ICG demonstrated excellent antibacterial efficacy against biofilms on Ti implant surfaces under the combined effects of PDT and sonodynamic therapy (SDT) [65]. Yuan et al. [66] employed mesoporous polydopamine nanoparticles (MPDA NPs) as a carrier for ICG, which was subsequently coupled with osteogenic RGD peptides (a tripeptide composed of arginine–glycine–aspartic acid) on the surface via Michael addition and Schiff base reactions. The integration of NIR-triggered photothermal therapy (PTT) and PDT demonstrated synergistic antibacterial effects, wherein elevated temperatures initially facilitated ICG diffusion into the biofilm, and the ROS produced by PDT compromised bacterial membranes, enhancing their susceptibility to moderate heat. This method expedited bacterial eradication with an in vivo efficiency of 95.4%, while RGD peptides also enhanced bone integration efficiently (Fig. 3B and C). The use of osteogenic active peptides offers a strategic pathway toward multifunctional implant fabrication.

Cyanine 7 (Cy7), a derivative of ICG, demonstrates peak light absorption in the NIR region. Nonetheless, the limitations of Cy7, including hydrophobicity, rapid photobleaching, and inadequate ROS production, restrict its further utilization [63]. Recent investigations indicate that the halogenation of cyanine dyes can markedly improve radical yields via heavy atom effects [67]. Halogenated anthraquinone dyes, shown by IR780, a novel iodine-substituted Cy7, have superior optical stability and augmented mitochondrial targeting capabilities relative to ICG [68]. Tan et al. [69] fixed positively charged IR780 onto negatively charged red phosphorus (RP) fiber membranes using electrostatic adsorption and subsequently modified the RP membranes with polydopamine (PDA) to incorporate osteogenic peptides (RGDC). Under 808-nm NIR irradiation, the ^1^O₂ produced by IR780 markedly increased the temperature sensitivity of *S. aureus* biofilms, resulting in effective biofilm eradication both in vitro and in vivo through the synergistic effects of 50 °C PTT (induced by RP) and PDT (mediated by IR780) while preserving normal tissues. The experimental findings indicated that the in vivo antibacterial efficacy attained 96.2%. Although the short-term effects of NIR irradiation did not harm the surrounding tissue in vivo, a notable reduction in MC3T3-E1 cell viability was observed in vitro owing to the adverse effects of the bacteria, heat, and ROS. The modified RGDC peptides were shown to promote the activities of MC3T3-E1 osteoblasts in vitro; however, the absence of in vivo osseointegration assessment undermines the utility of this investigation as a valuable reference for subsequent studies.

#### Natural organic photosensitizers

Curcumin, a yellow polyphenolic pigment derived from turmeric rhizomes, absorbs light in the 420- to 460-nm spectrum and exhibits multiple biological properties, including antibacterial, antioxidant, anti-inflammatory, and anticancer effects. As a natural photosensitizer, curcumin provides benefits like affordability, safety, high efficacy, and negligible side effects [70]. Numerous studies have shown that curcumin’s photodynamic properties can suppress the proliferation of *Enterococcus faecalis*, *Escherichia coli*, and *S. aureus* [71]. In an in vitro study, Guo et al. [70] utilized the superior adhesive characteristics of PDA to immobilize curcumin (Cur) onto Ti surfaces, resulting in Ti-PDA-Cur composites that efficiently eradicated *S. aureus* and *E. coli* via the production of ^1^O₂ and the photothermal effect of PDA under dual light irradiation (405 + 808 nm). Although curcumin effectively absorbs the 405-nm laser, this visible light, which can be partially absorbed by hemoglobin in the blood, possesses a very limited penetration depth, thereby impairing its efficacy in addressing deep-seated implant infections. Consequently, further investigations are urgently required to evaluate its in vivo therapeutic effectiveness. A separate investigation involved the development of curcumin-conjugated upconversion nanoparticles (UCNPs) to address this issue. UCNPs absorb NIR light and convert it into higher-energy visible light to facilitate the light absorption of curcumin. Due to the combined antibacterial behavior of UCNP–curcumin conjugates and PDT, nearly 100% of MRSA was eradicated under NIR irradiation in vitro. The in vivo periprosthetic joint infection model also achieved about 80% of MRSA elimination [72].

**Fig. 4.**
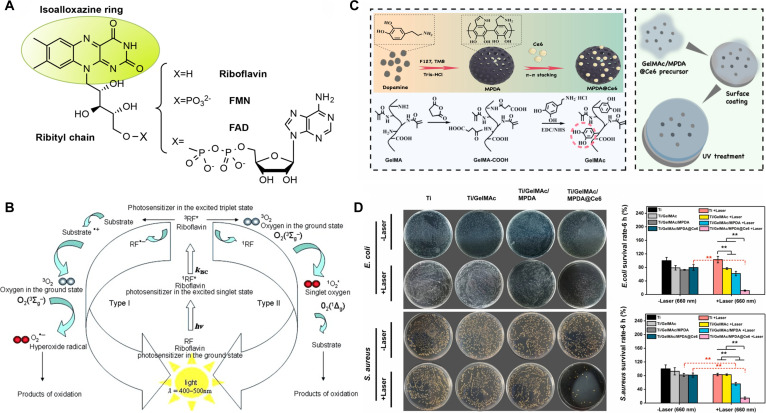
(A) Structures of riboflavin (RF), flavin adenine dinucleotide (FAD), and flavin mononucleotide (FMN). Reproduced with permission [74]. Copyright 2016, Elsevier. (B) Scheme of photosensitization reaction occurring with riboflavin as photosensitizer. Reproduced with permission [73]. Copyright 2014, Wiley. (C) Schematic illustration of the preparation of the GelMAc@MPDA@Ce6 hydrogel coating on titanium surfaces. (D) In vitro antibacterial evaluations of Ti/GelMAc/MPDA@Ce6. Reproduced with permission [79]. Copyright 2021, Elsevier.

Riboflavin, a water-soluble vitamin, possesses an isoalloxazine ring structure that is intrinsically linked to its ultraviolet (UV) absorption, fluorescence, redox characteristics, and photosensitivity. Upon exposure to visible or UV-A light, the isoalloxazine ring transitions to a singlet state exhibiting intense fluorescence, which then decays via ISC to a highly reactive and persistent triplet excited state. The latter is a potent oxidant that can oxidize a wide range of biomolecules via type I reactions and generate electrophilic ^1^O₂ through type II reactions [73,74] (Fig. 4A and B). Research indicates that riboflavin and its derivative flavin mononucleotide (FMN) can effectively diminish *S. aureus* biofilms on Ti implants when subjected to 445-nm laser and high-power blue light irradiation, demonstrating the antibacterial efficacy comparable to traditional MB treatments [75,76]. Compared to other dyes that function as photosensitizers for dental implants, FMN has a cosmetic advantage because of its light yellow color and ease of removal. However, the FMN-based PDT treatment is only recommended as an adjunct to mechanical cleaning of implant surfaces, since it cannot eliminate all microorganisms and organic material. The limitation of the blue light-emitting diode’s (LED) poor penetration ability also restricts its further application.

Chlorin e6 (Ce6), a natural compound resulting from chlorophyll breakdown, is a second-generation photosensitizer characterized by an elevated ^1^O₂ yield, reduced skin retention, and diminished phototoxic side effects [77,78]. Due to its hydrophobic properties, Ce6 was loaded onto MPDA NPs using a π–π stacking reaction and subsequently integrated into a multifunctional catechol motif-modified gelatin methacrylate (GelMA) hydrogel coating to ensure strong adhesion to Ti surfaces. Under a 660-nm laser (1 W/cm^2^), GelMAc/MPDA@Ce6 demonstrated efficient antibacterial efficacy, with only 11.45% of *E. coli* and 14.40% of *S. aureus* showing the capacity for survival (Fig. 4C and D). Following bacterial eradication, the down-regulated low-power laser irradiation (100 mW/cm^2^) exerted photobiomodulation effects, expediting wound healing through the activation of fibroblasts. The integration of PDT with low-level laser therapy (LLLT) successfully achieved a versatile transition between antibacterial and tissue healing activities by the modulation of irradiation power [79]. Although the adhesive hydrogel coating is suitable for the surface modification of most medical metal materials, more efforts are still needed to evaluate the physical properties of such hydrogel coating, including stiffness change, mechanical strength, and degradation control, which are vital for a successful integration between materials and natural tissues.

### Inorganic photosensitizers

Despite notable advancements in organic photosensitizers, their photostability and extinction coefficient do not adequately satisfy the demands of PDT. Moreover, their hydrophobic characteristics and inadequate stability pose obstacles for practical applications. Compared to organic materials, inorganic photosensitizers demonstrate extended durability, enhanced photostability, and functionalization potential, garnering increasing interest.

Semiconductor materials are regarded as highly promising inorganic photosensitizers owing to their excellent photocatalytic activity, stability, and cost-effectiveness. Based on the differential concentration of charge carriers, semiconductors are typically classified as p-type or n-type. The band structure of semiconductors, encompassing the positions of the valence band (VB) and conduction band (CB), as well as the band gap, dictates their light absorption characteristics and the redox capabilities of photo-induced charge carriers. When photon energy surpasses the semiconductor’s absorption threshold, electrons in the VB experience an interband transition to the CB, thereby generating electrons (e^−^) in the CB and leaving holes (h^+^) in the VB. Photo-induced electron-hole pairs can migrate to the surface, where they engage in redox reactions with adsorbed water and oxygen, resulting in the formation of ROS, including ·OH, O_2_^−^, and H_2_O_2_. Alternatively, the electron-hole pairs may be sequestered by defect sites or recombined, releasing energy as heat or light [31,80,81].

To date, metal semiconductors and carbon-based nanomaterials with distinctive optical and electrical characteristics have been extensively utilized in PDT. However, single-component inorganic photosensitizers often face substantial limitations that impede their application on Ti implants: narrow or negligible absorption in visible/NIR spectra owing to wide band gaps, inefficient photoinduced carrier separation, and rapid charge recombination [81]. To overcome these constraints, constructing semiconductor heterojunction composites (e.g., P–N heterojunctions, Schottky heterojunctions, and Z-scheme systems) with tailored band gaps has emerged as a prevalent strategy to enhance carrier separation efficiency and amplify PDT outcomes. Furthermore, defect engineering, particularly oxygen vacancy introduction, serves as an effective method for performance improvement. As the predominant anionic vacancy defect, optimal oxygen vacancy concentrations facilitate carrier transport modulation, increase charge carrier density, and provide abundant oxygen adsorption sites, thereby promoting ROS generation [31,81]. These strategies enable robust PDT implementation via inorganic photosensitizers at Ti implant interfaces.

#### Ti dioxide

**Fig. 5.**
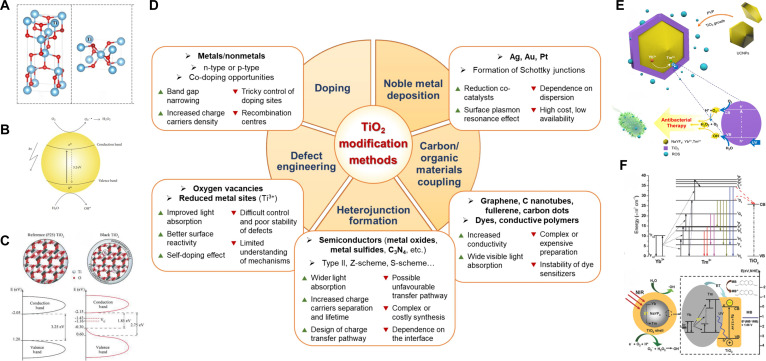
(A) Crystalline forms of TiO_2_, anatase, and rutile. Small spheres and large spheres represent O^2−^ and Ti^4+^ ions, respectively. Reproduced with permission [82]. Copyright 2016, Springer. (B) Schematic of ROS generation under irradiation of semiconducting TiO_2_ NP. Reproduced with permission [83]. Copyright 2012, Wiley. (C) Schematic of the nanoparticle’s structure and density of states (DOS) for TiO_2_ P25, Degussa, and black TiO_2_. Reproduced with permission [217]. Copyright 2012, American Chemical Society. (D) Main modification methods for obtaining TiO_2_-based materials with enhanced photocatalytic/photodynamic properties. Reproduced with permission [143]. Copyright 2024, Elsevier. (E) Schematic illustration of UCNPs@TiO_2_ enabling aPDT under NIR irradiation. Reproduced with permission [218]. Copyright 2019, Elsevier. (F) Illustrative diagrams of energy transfer among Yb^3+^, Tm^3+^, and TiO_2._ Reproduced with permission [219]. Copyright 2013, American Chemical Society.

Ti dioxide (TiO_2_) is an inorganic photosensitizer characterized by exceptional biocompatibility and enduring stability inside biological systems. It has received approval from the U.S. FDA for implantation use [1]. As a conventional n-type semiconductor, TiO_2_ has a band structure consisting of a filled low-energy VB and an unoccupied high-energy CB. Nonetheless, pure TiO_2_ possesses a broad bandgap (3.0 to 3.2 eV), permitting absorption solely of UV radiation (Fig. 5A and B) [82,83]. Owing to the limited tissue penetration depth of UV light and the obstruction of covered tissues, UV-excited TiO_2_ treatment modalities are incapable of providing efficient deep-tissue phototherapy [80]. Moreover, it is widely documented that UV radiation is detrimental to human health, presenting hazards when subjected to extended exposure. In contrast, NIR light (700 to 1,100 nm) conveys lower energy for therapeutic applications, inflicts minimal damage to healthy tissues, and achieves enhanced tissue penetration depth [84], consequently constituting an ideal irradiation source for the phototherapy of Ti implants. Two viable strategies have been suggested to fabricate NIR-activated TiO_2_ [80]: (a) employing UCNPs as nanotransducers to facilitate efficient energy conversion from NIR to UV light; (b) altering the optical absorption characteristics of TiO_2_ through nanostructural design, doping with metallic or nonmetallic elements, and coupling with other semiconductor materials. These methods not only broaden its absorption spectrum into the NIR region but also markedly enhance the photodynamic properties of TiO_2_ (Fig. 5D).

**Fig. 6.**
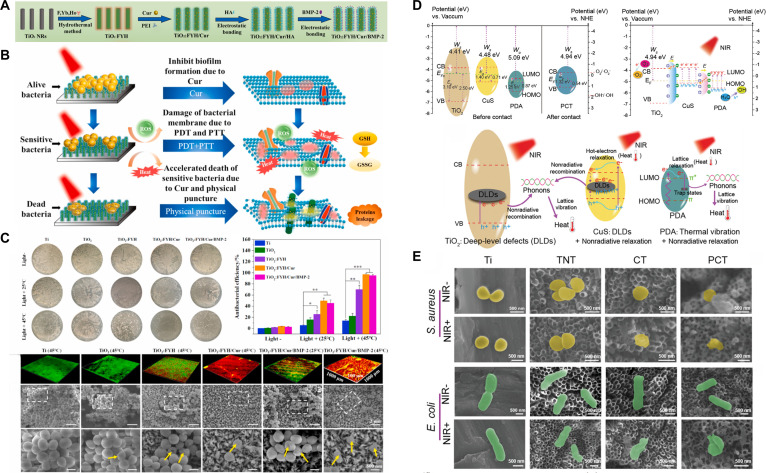
(A) Schematic illustration of the crafting process of the TiO_2_:FYH/Cur/BMP-2 NRs on Ti implants. (B) Biofilm elimination mechanism of TiO2:FYH/Cur/BMP-2 NRs under 1,060-nm NIR-II light irradiation based on quorum-sensing inhibitors (QSIs), PDT, mild-temperature PTT, and physical puncture. (C) Antibacterial activity of TiO_2_, TiO_2_:FYH, TiO_2_:FYH/Cur, and TiO_2_:FYH/Cur/BMP-2 NRs in vitro. Reproduced with permission [86]. Copyright 2021, Elsevier. (D) Photodynamic and photothermal mechanisms of PDA/CuS/TiO_2_ (PCT) under NIR irradiation. (E) Morphological changes of *S. aureus* and *E. coli* on PCT surfaces. Reproduced with permission [93]. Copyright 2022, Wiley.

UCNPs are widely utilized as fluorescent biological probes and exhibit advantages including narrow emission peaks, high quantum yield, excellent photostability, prolonged lifetime, and low toxicity. In UCNP-assisted upconversion photochemical processes, NIR light is transformed into UV–visible light, thereby activating the photoreactions of photosensitizers [85]. Among various UCNPs, NaYF_4_/:NaGdF_4_:Yb~(3+)/Tm~(3+) is an optimal selection, whose conjugation with TiO_2_ broadens the light absorption spectrum and facilitates efficient PDT under NIR irradiation (Fig. 5E and F) [80]. Furthermore, the incorporation of rare earth ions into TiO_2_ can produce analogous upconversion effects. Zhang et al. [86] co-doped TiO_2_ nanorods with F, Yb, and Ho elements via a hydrothermal technique, imparting TiO_2_:FYH (F, Yb, Ho) implants with upconversion capabilities and improved photodynamic performance. Meanwhile, curcumin and BMP-2 were grafted onto the surface successively via electrostatic binding to mitigate the inflammation and promote new bone formation. The resulting TiO_2_ nanorods responded to 1,060-nm NIR-II lasers, generating a large amount of ·OH and O_2_·^−^ radicals to collaboratively eliminate *S. aureus* biofilms. The nanorods attained a 95.2% antibacterial efficacy at a moderate temperature of 45 °C, facilitated by the combined actions of photogenerated ROS, curcumin’s quorum-sensing inhibition, and physical puncturing (Fig. 6A to C).

Despite their suitability as light converters, the limited conversion efficiency of UCNPs implies that high-power NIR lasers employed in UCNP-assisted phototherapy could harm normal skin and tissues. An optimal and efficient strategy is to modify the light absorption spectrum of TiO_2_ to encompass the NIR region. Multiple investigations have demonstrated that doping TiO_2_ with diverse metal and nonmetal elements can improve its light absorption in the visible and NIR areas [87]. Wu et al. [88] synthesized fluorine-doped TiO_2_ nanoparticles on Ti implants using a hydrothermal technique. The introduction of F ions improved TiO_2_’s light absorption, electron-hole separation, and crystallinity. Under 808-nm laser irradiation, F-TiO_2_ nanostructures could be elevated to an adequate temperature and produce sufficient ROS, leading to the total eradication of osteosarcoma cells after a mere 10 min. Meanwhile, the PDA–collagen coating onto the F-TiO_2_ further enhanced the proliferation and osteogenic differentiation of bone marrow mesenchymal stem cells (BMSCs) without influencing its photodynamic performance. In addition, Chi et al. [89] developed an oxygen vacancy-rich polydopamine/TiCu nanocoating (PDA/p-TiCu-300 °C) enabling multimodal photothermal/photodynamic/sonodynamic therapy. The doped Cu and oxygen vacancies greatly enhanced the photothermal effect and ROS generation under NIR light and ultrasound irradiation. Through integration of triple bactericidal mechanisms, including PTT, PDT, and SDT, this coating exhibited efficient disinfection against pathogenic bacteria. Another study conducted by Wu et al. [90] demonstrated that the introduction of Ti^3+^ and oxygen vacancies under high temperature and pressure markedly improved the photogenerated charge carrier transfer and photothermal conversion efficiency while shifting the light absorption to the NIR range. The resultant titanate/TiO_2−*x*_ heterostructures exhibited moderate photothermal effects and notable photodynamic efficacy, achieving 98.78% effectiveness against *S. aureus* and 98.33% against *E. coli.* Meanwhile, the highly porous micro/nanocoatings that mimic the natural bone extracellular matrix (ECM) showed exceptional osteogenic potential.

The effects of ion doping in TiO_2_ are mostly contingent upon the kind and quantity of ions; however, the actual doping amount is limited and does not exhibit a linear correlation with the increase of optical characteristics [91]. In contrast to ion doping, the formation of heterojunctions between TiO_2_ and other low-bandgap semiconductors offers greater flexibility and control, allowing effective NIR light absorption while markedly enhancing the dissociation of electron-hole pairs. Various agents, including noble metal nanoparticles, metal oxides, metal sulfides, and carbon nanomaterials, have been reported to produce heterojunctions with TiO_2_ [87,92]. Ding et al. [93] developed an innovative photoresponsive bio-heterojunction [PDA/CuS/TiO_2_ (PCT)] on Ti implants to mitigate recurring infections in percutaneous implants. This structure comprised TiO_2_ nanotubes infused with CuS NPs and encased in a PDA layer. An intrinsic electric field was established within PCT, directed from TiO_2_ to CuS and then to PDA, as a result of the Fermi level equilibrium. Under NIR irradiation, this electric field induced the migration of photo-excited electrons in opposing directions, resulting in the segregation of electron-hole pairs, thereby generating substantial quantities of ROS. The combined effects of hyperthermia and photogenerated ROS enhanced bacterial membrane permeability and cellular component leakage, granting PCT superior antibacterial efficacy. In a reinfection model, PCT not only eradicated germs and diminished inflammation but also improved soft tissue reintegration (Fig. 6D and E). In a separate investigation, Chen et al. [94] established a P–N heterojunction between the p-type semiconductor Co_3_O_4_ and n-type TiO_2_, markedly diminishing the recombination rate of electron-hole pairs. Furthermore, the CB potential of the P–N heterojunction was synchronized with the biological redox potential, establishing an electron transfer pathway with bacteria that interfered with their internal electron transport, resulting in elevated intracellular ROS. The Co_3_O_4_/TiO_2_-Ti implants demonstrated exceptional antibacterial efficacy, eradicating 95.71% of both planktonic and adhering *S. aureus* in vitro.

Following the initial synthesis of black hydrogenated TiO_2_ (H-TiO_2_) from white anatase TiO_2_ by Chen et al. [95] using hydrogen reduction, H-TiO_2_ has garnered substantial research interest owing to its distinctive optical characteristics. In contrast to TiO_2_, the alterations in the crystal structure and chemical composition of H-TiO_2_, such as the emergence of midgap states and the movement of the VB and/or CB, result in a substantial reduction of its bandgap and a redshift in light absorption, enabling its response to NIR light stimulation [80]. Besides, the narrow bandgap and plentiful oxygen vacancies endow H-TiO_2_ with superior photothermal and photoelectric conversion properties, making it an ideal candidate for effective PTT and PDT (Fig. 5C) [80]. At present, 2 primary techniques for synthesizing H-TiO_2_ have been documented: reduction methods and high-energy treatment methods exemplified by ultrasound and pulsed laser treatment. Reduction methods are the most prevalent due to their simplicity and efficiency, encompassing chemical reduction techniques utilizing reducing agents such as hydrogen, hydrogen plasma, aluminum, magnesium, NaBH_4_, and NaH as well as electrochemical reduction [96]. Guo et al. [97] synthesized mesoporous H-TiO_2_ spheres through hydrogen reduction, which demonstrated effective absorption in the NIR region and functioned in 3 capacities (PDT photosensitizer, PTT agent, and doxorubicin carrier) in trimodal chemical/photodynamic/photothermal therapy. In a separate investigation, Chen et al. [98] developed a “photo-thermal-electric” platform for anti-infection and bone immunoregulatory implants. This platform showcased ZnO/black TiO_2−*x*_ nanofilms that created abundant oxygen vacancies and established heterojunction structures, thereby markedly augmenting the photothermal and photoelectric performance under NIR illumination. The photo-thermal-electric implant showed remarkable broad-spectrum antibacterial effectiveness against 3 tooth infections by compromising bacterial membranes and enhancing intracellular ROS generation. Furthermore, by establishing a conducive osteoimmune milieu and facilitating the formation of pro-restorative M2 macrophages, the implant promoted osseointegration both in vitro and in vivo. The activated photocurrent plays an important role in both antibacterial and osteoimmunomodulatory activities of this implant, but its specific mechanism is not clearly elucidated and requires further investigation.

Driven by advances in nanotechnology, a crucial approach involves engineering the TiO_2_ structure and surface at the nanoscale. Nano-TiO_2_ can manifest in various dimensions and configurations, including TiO_2_ nanorods, nanotubes, nanoneedles, nanopillars, nanopores, and nanowires [99]. These diverse morphologies not only improve their photodynamic and photothermal performance but also offer appropriate platforms for the incorporation of additional materials to boost antibacterial activity. TiO_2_ nanotubes or nanopores constructed on Ti surfaces can serve as vehicles for antibacterial compounds [100]. TiO_2_ nanorod or nanoneedle arrays can kill bacteria by mechanically compromising their membranes and causing the release of intracellular contents [101]. Simultaneously, the established hierarchical structures can augment the light-harvesting capacity and anti-reflective effectiveness of the metallic surface, leading to improved photothermal and photodynamic effects [102]. Importantly, hierarchical patterns on Ti surfaces can replicate the natural ECM, enhancing fluid flow and molecular transport while boosting osteogenic signaling, hence improving cell adhesion, migration, proliferation, and differentiation [103,104]. Zhang et al. [102] developed an ordered TiO_2_ nanorod array through advanced structural design, which accomplished a multifaceted antibacterial mechanism integrating PTT, PDT, and physical puncturing under 808-nm NIR irradiation, without the requirement for additional photosensitizers or the release of other ions. The biofilm infection was effectively eliminated through multiple antibacterial actions, while the TiO_2_ nanorod arrays facilitated osteoblast proliferation, spreading, and differentiation, thereby substantially enhancing new bone formation even within an infected microenvironment in vivo.

**Fig. 7.**
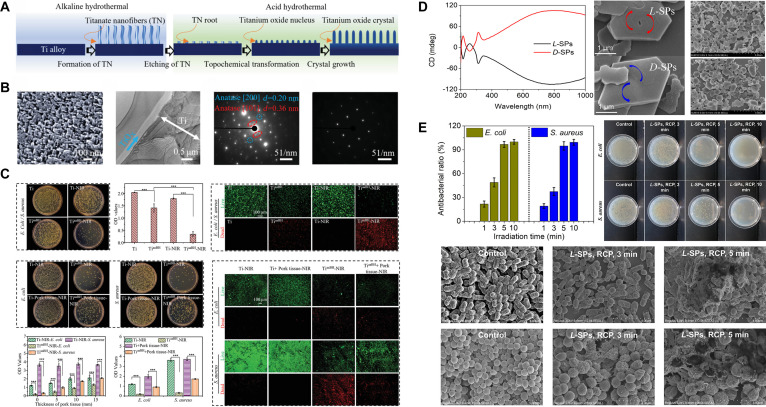
(A) The general design principle of the aaBH method is to construct a quasi-periodic titanium oxide metasurface on Ti implants. (B) From left to right: Scanning electron microscopy (SEM) image of the Ti^aaBH^, transmission electron microscopy (TEM) images of the cross-section of Ti^aaBH^, diffraction pattern of Ti^aaBH^, and diffraction pattern of Ti. (C) In vitro antibacterial property of Ti and Ti^aaBH^. Reproduced with permission [106]. Copyright 2021, Wiley. (D) CD spectra and SEM images of chiral TiO_2_ SPs. (E) Circularly polarized light (CPL)-driven photodynamic antibacterial activity of L-SPs. Reproduced with permission [108]. Copyright 2023, ACS Publications.

The metasurface is a 2D artificial optical nanostructure composed of subwavelength-scale unit elements, engineered to precisely manipulate the amplitude, phase, polarization, and other characteristics of light waves [105]. Recent investigations have shown that sophisticatedly designed metasurface-nanostructured TiO_2_ exhibits superior light manipulation capabilities. Yang et al. [106] successfully fabricated a TiO_2_/TiO_2−*x*_ metasurface exhibiting robust NIR-responsive antibacterial properties on Ti alloy implants by an alkaline-acid bidirectional hydrothermal technique (aaBH). The dimensions and sizes of the nanostructural units in the metasurface were extremely adjustable, facilitating versatile modulation of light absorption. Exposed to low-power NIR light for 10 min, the TiO_2_/TiO_2−*x*_ metasurface generated substantial quantities of ^1^O₂ and ·OH, demonstrating remarkable antibacterial efficacy against *E. coli* and *S. aureus* (Fig. 7A to C). Although there was no evaluation of osseointegration efficacy, both in vitro and in vivo studies demonstrated its excellent biocompatibility. The facile aaBH treatment can yield huge functional improvement, highlighting the promising potential of nanoscale topography manipulation. Wu et al. [107] employed the same methodology to fabricate a light-responsive TiO_2_ metasurface and thoroughly examined several process parameters. The findings demonstrated that hydrochloric acid (HCl) and post-heat-treatment promoted redshift in light absorption. Alongside exceptional photodynamic antibacterial efficacy, the resultant TiO_2_/TiO_2−*x*_ metasurface enhanced cell adhesion, proliferation, differentiation, and the expression of osteogenic-related genes. Surface chiral alterations of nanomaterials provide an alternate approach to attain a redshift in light absorption. Gao et al. [108] conducted surface chiral functionalization of TiO_2_ to modify its bandgap width, resulting in a wide circular dichroism (CD) absorption at 792 nm. The chiroptical activity of TiO_2_ superparticles (SPs) originated from atomic-scale distortions in TiO_2_ NPs and the helical patterns at the microscale in TiO_2_ SPs, exhibiting counterclockwise or clockwise orientations, respectively. By altering the orientation of circularly polarized light (CPL), they aligned the photon spin polarization direction with the valence electron transition direction of chiral TiO_2_ SPs, thereby facilitating the effective separation of electron-hole pairs. Under 808-nm CPL, ·OH and ^1^O₂ were produced efficiently, achieving 99.4% inhibition of Gram-positive bacteria and 100% inhibition of Gram-negative bacteria (Fig. 7D and E). The chiral TiO_2_ exhibits robust antibacterial activities and superior biocompatibility, providing a novel direction for the development and utilization of chiral nanomaterials with NIR absorbance in antibacterial medical implants.

In summary, TiO_2_, the FDA-approved implant material with extensive clinical utilization, manifests compelling potential as an ideal photosensitizer for photodynamic antibacterial therapy via appropriate modification. Diverse techniques have been established to fabricate NIR-excited TiO_2_ and augment its photodynamic efficacy. The hydrothermal method stands as the most prevalent methodology, enabling precise control over TiO_2_ elemental doping, metastructure formation, surface nanostructuring, and heterojunction construction. Nevertheless, this approach imposes stringent processing requirements, necessitating exact parameter optimization to achieve desired outcomes, thus presenting great challenges for scalable manufacturing. Some physical techniques, such as successive ionic layer adsorption and reaction (SILAR) and plasma-enhanced chemical vapor deposition (PECVD), provide simple, convenient, and cost-effective alternatives. However, PDT systems fabricated via these methods exhibit inherent stability limitations due to delamination risks. Therefore, to advance clinical translation of modified TiO_2_, future research should not only prioritize PDT efficiency and antibacterial efficacy but also focus on fabrication complexity, long-term stability, and cost-effectiveness—critical factors determining laboratory-to-clinic transition.

#### Carbon-based nanomaterials

Carbon, one of the most prevalent elements in nature, has been extensively utilized across multiple domains. Carbon-based nanomaterials, including carbon dots (CDs), carbon nanotubes (CNTs), graphene-based nanomaterials, and carbon nitride, have garnered heightened interest in the field of phototherapy owing to their distinctive features.

CDs have garnered massive attention owing to their biocompatibility, low cytotoxicity, NIR light absorption, and effective electron transfer capabilities [109]. CDs predominantly consist of sp^2^ carbon, which might impede electron-hole recombination and result in improved photodynamic effects [110]. Furthermore, CDs, as remarkable photothermal agents, have been highlighted [109]. He et al. [111] fabricated Ti implants by including CDs into the TiO_2_ nanorod array (C-TiO_2_ NR) to facilitate interfacial charge transfer. Under light excitation, electrons in the highest occupied molecular orbital (HOMO) of CDs were promoted to the lowest unoccupied molecular orbital (LUMO) and subsequently transported to the CB of TiO_2_, resulting in the formation of holes in the VB of CDs. These holes then interacted with OH^−^ to generate ·OH. Both the photothermal and photodynamic efficacy under dual-light irradiation at 808 and 660 nm were markedly improved, achieving an antibacterial effectiveness of 99.9% for C-TiO_2_ NR, compared to <20% for Ti. Huo et al. [112] developed a bismuth sulfide silver@carbon quantum dot composite coating (AgBiS₂@CQDs) on a medical Ti surface in a separate investigation. The utilization of CQDs as a charge transfer intermediary diminished the electron-hole recombination rate of the narrow-bandgap AgBiS₂, thereby improving photodynamic activity. Upon exposure to a 1,064-nm NIR laser, the AgBiS₂@CQDs/Ti efficiently catalyzed the production of ·OH and ^1^O₂ from water and oxygen in the coating’s vicinity, swiftly eliminating mature biofilms and pathogenic bacteria through the synergistic effects of PDT and PTT.

CNTs with ultrahigh specific surface area, diverse structures, and adjustable physicochemical properties, have garnered extensive attention in biomedical fields, such as drug delivery, biological imaging, bioscaffolds, and biosensors [113]. Recently, several studies have investigated the utilization of CNTs in the domain of photodynamic inactivation of microorganisms. Mohammad et al. [114] generated silver-doped TiO_2_ nanoparticle-modified CNTs via the sol–gel method. The use of CNTs diminished the bandgap of TiO_2_, facilitating the absorption of longer-wavelength light. Moreover, the excellent electron storage capacity of CNTs enabled them to receive photoinduced electrons from the CB of TiO_2_, thereby considerably augmenting the production of ROS. Zhu et al. [115] incorporated ZnO nanoparticles onto the surface of chitosan-modified CNTs by atomic layer deposition (ALD) to mitigate implant-associated infections. The CNTs impeded the recombination of photoinduced electron-hole pairs in ZnO, resulting in hybrid nanostructures that demonstrated improved photodynamic activity and high self-antibacterial efficiency, achieving clearance rates of *E. coli* and *S. aureus* exceeding 73% and 98%, respectively. Furthermore, by adjusting the cycling numbers of the ALD process, the Zn concentration could be modulated, hence facilitating the proliferation and osteogenic differentiation of osteoblasts. The ALD technique offers a facile way to precisely regulate ZnO content and avoid the possible cytotoxicity of excessive ZnO, deserving further application for engineering Ti implant surfaces.

Graphene oxide (GO) has emerged as a focal point of research due to its multifaceted antibacterial mechanisms, including oxidative stress induction, physical entrapment, membrane penetration via sharp edges, and photothermal/photodynamic effects [116]. Furthermore, oxygen-functional groups on GO’s backbone facilitate critical biomolecular interaction, such as protein adsorption, cell adhesion, proliferation and differentiation, Ca^2+^ binding, and bone matrix mineralization, establishing its functionality in bone tissue engineering [117]. Owing to its superior conductivity, photoinduced electrons can rapidly move from the photocatalyst to GO, effectively suppressing electron-hole recombination. Meanwhile, GO modulates the band alignment of composite semiconductors, resulting in a redshift of light absorption [116]. Xie et al. [118] documented a PDA/Ag₃PO₄/GO nanohybrid coating, wherein GO adjusted the bandgap of Ag₃PO₄ from 2.52 eV to 2.0 eV, facilitating the generation of ROS under 660-nm visible light irradiation. The continuous release of Ag^+^ and the augmented photodynamic impact generated combined antibacterial outcomes, with mortality rates of *E. coli* and *S. aureus* reaching 99.53% and 99.66%, respectively. Li et al. [119] developed a heterojunction of hydroxyapatite/nitrogen-doped CD-modified graphene oxide (GO/NCD/Hap) on Ti surfaces. The incorporation of GO markedly facilitated the dissociation of photoinduced electron-hole pairs, leading to swift bacterial eradication via augmented photodynamic and photothermal effects following NIR light exposure. Moreover, the values of the bacterial respiration chain (4.1 to 4.8 eV), which exceeded the potential of the Ti film, facilitated the extraction of membrane electrons from the transmembrane protein complex of *S. aureus*. This phenomenon caused an interruption in the respiration chain and damage to the membrane structure, ultimately resulting in a combined antibacterial effect due to the excessive production of ROS and disrupted ATP synthesis. Furthermore, the electron transfer between the Ti film and cell membrane may elicit the Ca^2+^ influx, hence activating the phospholipase Cγ1 (PLCγ1)/extracellular signal-regulated kinase (ERK) pathway, which enhanced cell migration and proliferation, alkaline phosphatase (ALP) activity, and vascular damage repair. The resultant photocurrent also alleviated the inflammation induced by phototherapy via the activation of the phosphatidylinositol 3-kinase (PI3K)/P-AKT pathway. This gentle phototherapy strategy, utilizing photocurrents to concurrently achieve bacterial eradication, inflammation modulation, and tissue reconstruction, holds great potential for the clinical application of noninvasive phototherapy in the future.

**Fig. 8.**
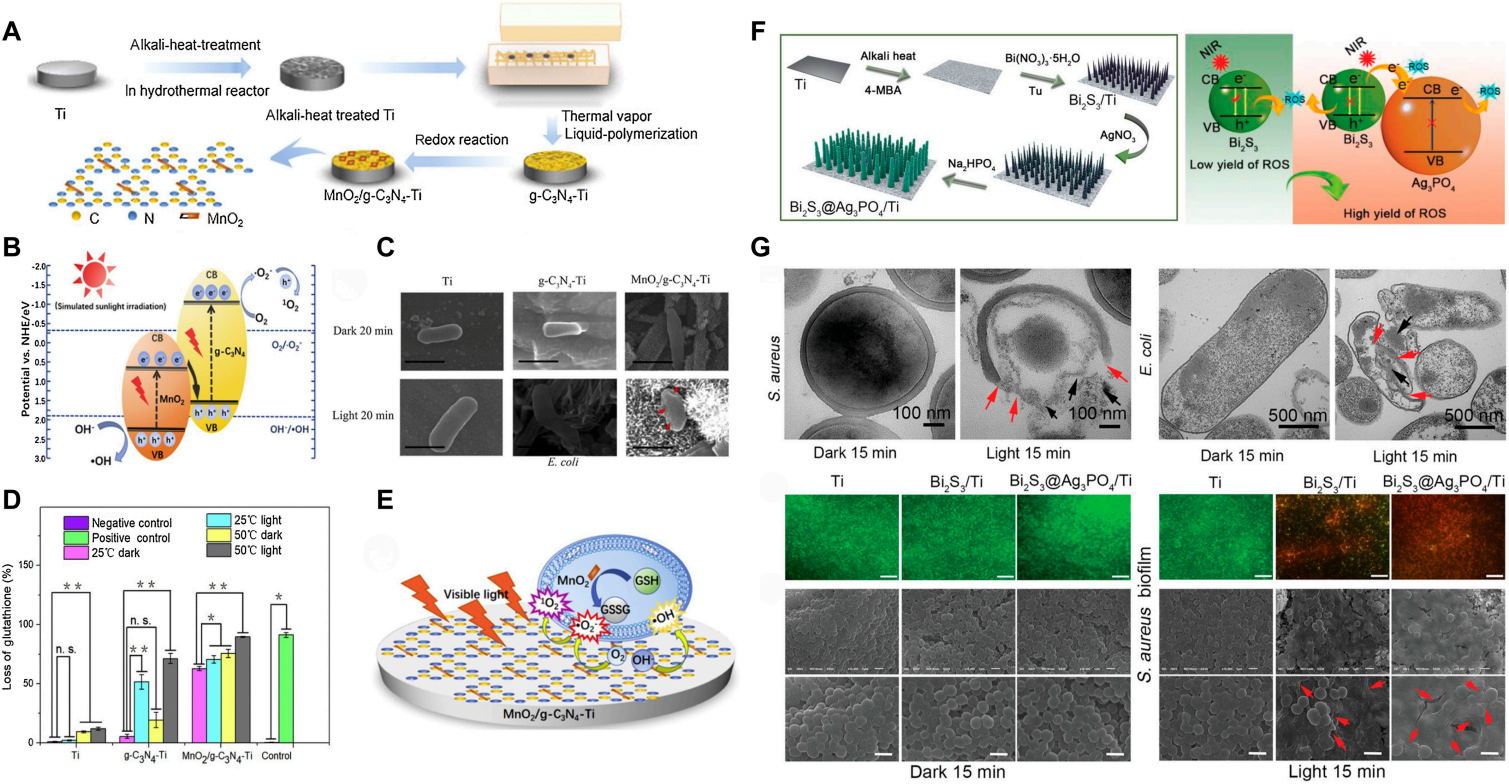
(A) Procedure of synthesizing MnO_2_/g-C_3_N_4_-Ti. (B) Scheme of the mechanism of photoenergy transfer on the MnO_2_/g-C_3_N_4_ heterostructure for generating ROS. (C) SEM morphology of *E. coli* on the surface of MnO_2_/g-C_3_N_4_-Ti. (D) GSH depletion enhanced by MnO_2_. (E) Schematic illustrating the mechanism of the antibacterial property of MnO_2_/g-C_3_N_4_-Ti under visible light. Reproduced with permission [122]. Copyright 2019, Elsevier. (F) Illustration for the synthesis procedures for Bi_2_S_3_@Ag_3_PO_4_ nanorod arrays on Ti plates. (G) In vitro antibacterial ability of Bi_2_S_3_@Ag_3_PO_4_/Ti. Reproduced with permission [134]. Copyright 2019, Wiley.

Carbon nitride (C₃N₄) is a carbon-derived semiconductor composed of plentiful carbon and nitrogen atoms. The distinctive attributes of C₃N₄, including exceptional photoelectric capabilities, superior biocompatibility, and facile production, have resulted in its extensive application in photodynamic treatment [120]. Nevertheless, the fast recombination of photogenerated electrons and holes in pure C₃N₄ greatly hinders the therapeutic action. An effective method for enhancement is integrating C₃N₄ with other semiconductors to form heterojunctions [121]. The formation of a Z-scheme heterojunction in proximity to MnO_2_ resulted in the MnO_2_/g-C₃N₄ heterojunction coating on Ti’s surface, which facilitated efficient separation of photoinduced electron-hole pairs, hence enhancing the photo-conversion efficiency of g-C₃N₄ by 21.11%. Upon exposure to sunlight, the photogenerated electrons in MnO_2_ swiftly migrated to g-C₃N₄, where they recombined with holes in the VB of g-C₃N₄, leading to abundant free electrons in the CB of g-C₃N₄ and corresponding holes in the VB of MnO_2_. Subsequently, these free electrons reacted with O₂ to produce O₂·^−^, whereas the holes contacted water molecules to generate ·OH. MnO_2_ not only augmented the photodynamic properties of g-C₃N₄ to elevate ROS yields but also diminished glutathione (GSH), which defends against oxidative stress in bacteria, hence enhancing the antibacterial efficacy. Following 20 min of visible light irradiation, the MnO_2_/g-C₃N₄ coating demonstrated exceptional disinfection effectiveness of 99.96% and 99.26% against *S. aureus* and *E. coli*, respectively (Fig. 8A to E) [122]. This coating, characterized by facile preparation, low cost, and good biocompatibility, is a potential candidate for the sterilization of medical implants. Beyond heterojunction engineering, recent studies have integrated functionalized groups (e.g., halogen, polyethyleneimine, and acridinium groups) with C₃N₄ to enhance bacterial interactions, yielding notable performance improvement [121,123].

#### Metallic elements and their compounds

Metals and metal compounds exhibit broad absorption spectra, enabling efficient light harvesting across wavelengths spanning UV to NIR regions. Diverse metal–semiconductor materials with multi-band structures can be engineered into versatile heterostructures, thus effectively mitigating high electron-hole recombination rates and promoting ROS generation during PDT [124]. Furthermore, the inherent rigidity of metallic components enhances the mechanical integrity of biomedical implants. These attributes establish metal-based PDT as a promising choice for clinical Ti implants. To date, extensive research in photodynamic antibacterial applications has encompassed various metallic materials, such as noble metals, metal oxides, metal sulfides, and MXenes.

##### Metal oxides

TiO_2_ has become the most extensively utilized photosensitizer due to its facile in situ fabrication on Ti implant surfaces, enabling diverse topological features and heterostructures. Various modification strategies have been detailed in preceding sections. Beyond TiO_2_, recent studies have explored the potential of other metal oxides to serve as photosensitizers. ZnO is a widely utilized antibacterial agent and has received approval from the U.S. FDA [125]. The activation of conventional ZnO necessitates detrimental UV light sources. Zhao et al. [125] synthesized Tremella-like ZnO with a reduced energy gap compared to ordinary ZnO, enabling activation by benign visible yellow light. Furthermore, the Tremella-like ZnO demonstrated enhanced photothermal conversion efficiency when exposed to NIR light. The dual-light-sensitive ZnO@Collagen-I composite coating on the Ti surface enhanced osteogenesis and expedited osseointegration through the combined effects of moderate heating and loaded collagen-I while simultaneously preserving superior broad-spectrum antibacterial efficacy via PDT. With the adjustment of double light (yellow and NIR light), this unique dual-functional coating well meets the demand of an ideal implant for both antibacterial and osteogenic activities, but the long-term stability of the coating needs to be confirmed by further experiments. In another study, a light-activated self-propelling ZnO:Ag micromotor was created to address bacterial biofilm infections. Upon exposure to UV light, photoinduced electrons produced by ZnO could migrate to the Ag component, leaving holes in the ZnO region, therefore reducing the recombination rate of photogenerated electron-hole pairs. Subsequently, the electrons reacted with oxygen molecules to yield O₂·^−^, which might undergo further conversion into HOO·, while the holes in ZnO interacted with water to produce ·OH. The combined effect of Ag^+^ and ROS enabled a modest concentration (1 μg/ml) of ZnO:Ag micromotor to successfully eradicate bacterial biofilms [126]. Given the inherent cytotoxicity of ZnO and Ag, further research necessitates a comprehensive evaluation of their biocompatibility.

##### Metal sulfides

MoS₂, a conventional layered transition metal dichalcogenide, is among the most extensively researched metal photosensitizers in Ti implants. It demonstrates distinctive physical, optical, and electrical characteristics attributed to its ultrathin 2D atomic layer configuration and high specific surface area [127]. The bandgap of MoS₂ nanosheets is thickness-dependent. When exfoliated from bulk to monolayer form, MoS₂ undergoes an indirect-to-direct bandgap transition, ranging from 1.2 eV to 1.9 eV. This tunability enables its broad-spectrum light absorption spanning UV to NIR regions across different layered configurations [128]. Particularly, it exhibits strong NIR absorption, positioning it as a promising candidate for PTT and PDT [127]. To achieve enhanced performance, chitosan is frequently employed to prevent the reaggregation of exfoliated hydrophobic MoS₂ nanodispersions. Feng et al. [129] developed a chitosan-assisted MoS₂ (CS@MoS₂) hybrid coating in situ, imparting photodynamic and photothermal properties to metallic Ti implants. The concurrent short-term exposure to 660-nm visible light and 808-nm NIR light markedly augmented the bactericidal efficacy against *E. coli* and *S. aureus*, resulting in eradication rates of 99.84% and 99.65%, respectively. The utilization of dual-light sources offers a viable strategy for enhanced antibacterial efficacy by combining PDT and PTT. Zhu et al. [130] incorporated Ag NPs onto a chitosan-modified MoS₂ coating by UV light-induced reduction in another work. Silver’s rapid electron transfer capacity facilitated the swift movement of photo-induced electrons from MoS₂ to Ag NPs, hence diminishing the recombination rate of electron-hole pairs. This hybrid system demonstrated excellent photodynamic activity and effectively eradicated bacteria under 660-nm light irradiation. Nevertheless, the inherent biotoxicity of Ag NPs remains a critical concern. The potential cytotoxicity arising from sustained Ag^+^ leaching requires rigorous assessment.

Copper is a vital trace element in the human body, and biomaterials containing copper have demonstrated the ability to enhance angiogenesis, facilitate wound healing, and support the regeneration of bone and cartilage [131]. Copper sulfides exhibit robust NIR absorption, excellent photostability, and minimal toxicity, rendering them optimal candidates for PTT and PDT. Dai et al. [132] synthesized CuS nanoclusters exhibiting both photothermal and photodynamic properties. Upon exposure to 980-nm NIR light, these CuS nanoclusters efficiently eradicated adhered bacteria and inhibited bacterial regrowth via the heat and ROS produced. Wang et al. [131] produced PEG-modified Cu_9_S_8_ nanoparticles through a simple one-step wet chemical process. The dandelion-shaped Cu_9_S_8_ nanoparticles exhibited outstanding photothermal performance, achieving a photothermal conversion efficiency of 41.55% alongside commendable photodynamic effects. Under 808-nm NIR irradiation, Cu_9_S_8_ nanoparticles demonstrated remarkable antibacterial efficacy and antibiofilm properties against clinically isolated pathogenic *S. aureus* on Ti plates. Cu_2−*x*_S (0 < *x* < 1) is a substoichiometric compound comprising various copper-rich to sulfur-rich copper sulfide phases, which confer exceptional optoelectronic characteristics and robust NIR light absorption. Moreover, its band-bending action markedly enhances the spatiotemporal separation of photoinduced electron-hole pairs, while the localized surface plasmon resonance (LSPR) effect endows Cu_2−*x*_S with excellent photothermal properties [11]. By a sacrificial template and co-deposition method, a NIR-responsive antibacterial and antifouling coating (Ti-PEG-Cu_2−*x*_S) was effectively developed on the surface of Ti implants. This coating integrated “active attack” and “passive defense” antibacterial mechanisms, effectively preventing bacterial attachment and proliferation on implants through the synergistic effects of the sensitive photothermal/photodynamic properties of Cu_2−*x*_S nanohomogeneous junctions and the non-adhesive surface of PEG [11]. Recognizing that bacterial initial attachment constitutes the determinant step in biofilm formation, integrating non-adhesive surfaces with phototherapy presents a strategic approach for preemptive biofilm suppression on medical implants.

Bismuth sulfide (Bi_2_S_3_) is a promising photocatalyst characterized by excellent biocompatibility, elevated photoconductivity, and superior photothermal conversion efficiency. Owing to its narrow bandgap (1.3 to 1.7 eV), Bi_2_S_3_ can generate electrons and holes when exposed to NIR light. Nonetheless, Bi_2_S_3_ is infrequently utilized independently in photodynamic reactions due to the high recombination rate of photoinduced carriers [133]. Hong et al. [134] utilized a stepwise electrostatic adsorption method to load wide-bandgap semiconductor Ag_3_PO_4_ nanoparticles onto Bi_2_S_3_ nanorod arrays, resulting in the formation of a Bi_2_S_3_@ Ag_3_PO_4_ hybrid heterojunction coating on the surface of Ti implant. Given that the CB potentials of Ag_3_PO_4_ are inferior to those of Bi_2_S_3_, photogenerated electrons from Bi_2_S_3_ can be efficiently transported to Ag_3_PO_4_ and subsequently migrate to the surface, thereby facilitating the separation of photogenerated electron-hole pairs. The increased specific surface area of Ag_3_PO_4_ nanoparticles offered additional active sites, which further promoted the photocatalytic redox process. The produced coating demonstrated an improved photodynamic effect and a favorable photothermal effect under 808-nm light irradiation, resulting in swift biofilm eradication within 15 min (Fig. 8F and G). In comparison to pure Ti, the coating exhibited good cell viability and reduced harm to normal tissue and cells.

##### MXenes

**Fig. 9.**
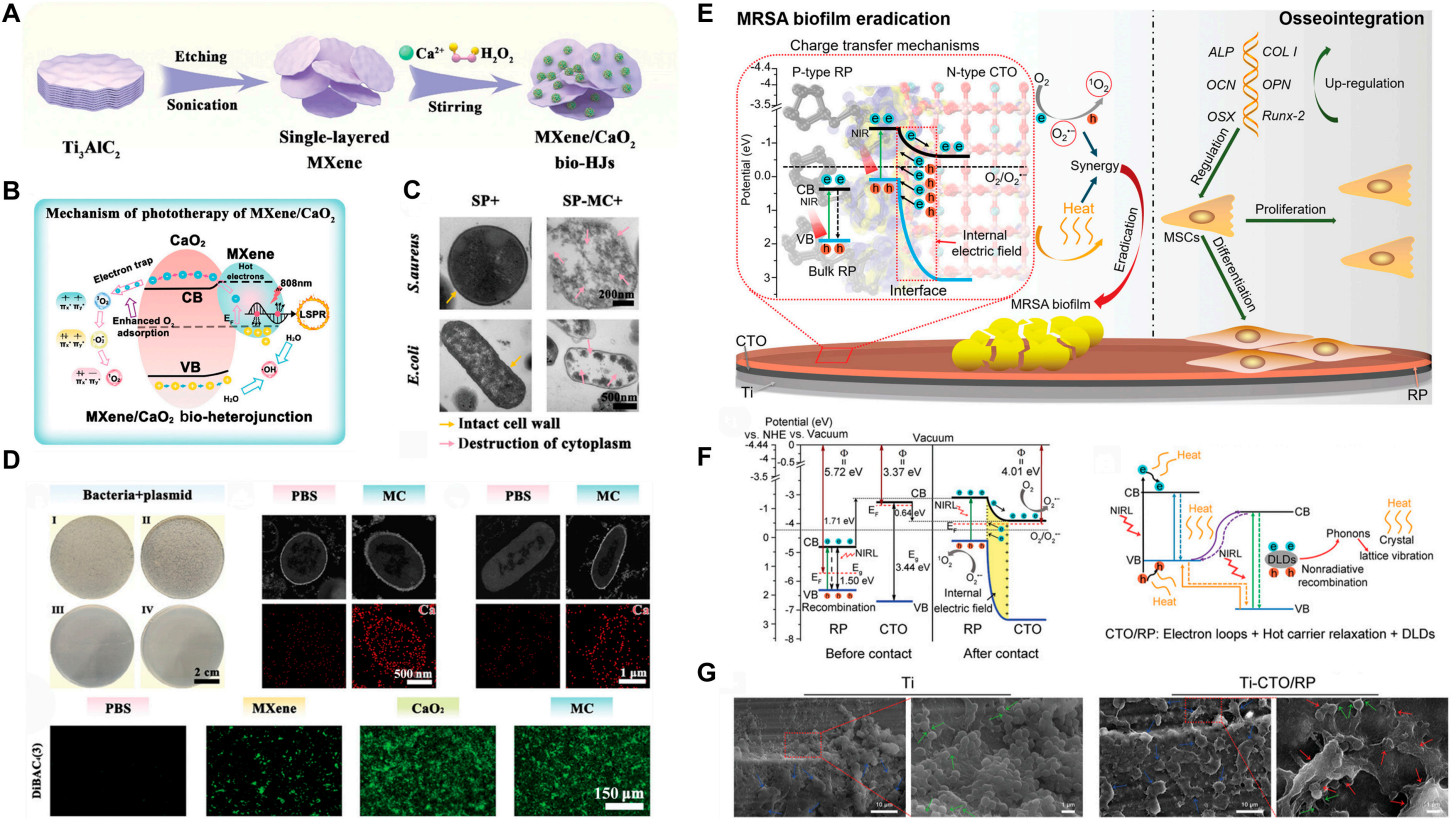
(A) Schematic preparation of MXene/CaO_2_ bio-heterojunction (MC bio-HJs). (B) Mechanism of phototherapy. (C) TEM images of *S. aureus* and *E. coli* after NIR irradiation. (D) Activation of a competent cell-like stage by Ca^2+^, as shown by the images of bacterial colonies of DH5α, the Ca element distribution in *S. aureus* and *E. coli*, and fluorescent images of DiBAC4. Reproduced with permission [137]. Copyright 2024, Wiley. (E) Schematic diagram of enhanced NIRL photodynamic eradication of MRSA biofilms and osseointegration of CTO/RP P–N heterojunction. (F) Schematic diagram of the photodynamic and photothermal mechanism of CTO/RP P–N heterojunction. (G) Representative SEM images of the MRSA biofilms on the surface of implants taken from rats at the end of treatments. Reproduced with permission [157]. Copyright 2021, Wiley.

MXenes, as flagship 2D transition metal carbides, nitrides, or carbonitrides, feature layered structures, intrinsic hydrophilicity, phototherapeutic properties, and tunable compositions characterized by the general formula M_*n*+1_X*_n_*T_x_ (*n* = 1 to 3). Here, M denotes early transition metals (e.g., Ti, V, Cr, Zr, Nb, and Mo), X represents carbon/nitrogen, and T_x_ signifies surface terminations (O, OH, F, Cl) that confer anisotropy and electric conductivity. These materials exhibit multimodal bactericidal mechanisms, such as physical membrane penetration via sharp edges, photothermal effects, and ROS generation, establishing them as promising non-antibiotic coating additives for orthopedic and dental implants [135]. Various deposition techniques, spanning drop casting, spin/soak/dip coating, layer-by-layer assembly, electrophoretic deposition, and 3D printing, enable their extensive application onto biomedical implant surfaces [136]. For their utilization in PDT, Huang et al. [137] engineered MXene (Ti_3_C_2_)/CaO₂ bio-heterojunctions (MC bio-HJs) via an in situ growth method, implementing a competent cell-like antibacterial strategy. This heterostructure formation induced a work function shift to 3.7 eV, intermediate between Ti_3_C_2_ (4.5 eV) and CaO₂ (2.6 eV), enabling spontaneous electron transfer from Ti_3_C_2_ to CaO₂. Consequently, electron enrichment occurred at CaO₂ surfaces while holes accumulated on Ti_3_C_2_. These electrons reacted with environmental O₂ to generate ^1^O₂, while holes oxidized H₂O to produce ·OH radicals. Simultaneously, Ca^2+^ coordination with bacterial membrane phospholipids potentiated membrane permeability. Under NIR irradiation, the tremendous heat and ROS generated by PTT and PDT of MC bio-HJs easily penetrated compromised membranes, accumulating intracellularly to cause irreversible structural and functional damage, leading to disrupted bacterial energy metabolism and protein synthesis. As a result, the engineered bio-HJs achieved near-complete eradication (≈100%) of both drug-resistant MRSA and common pathogens (Fig. 9A to D). When coated on orthopedic implants, these heterostructures also demonstrated satisfactory osseointegration enhancement in infected bone defects. Zhu et al. [138] fabricated MXene/Ag₃PO₄-glucose oxidase bio-heterojunctions (MX/AgP-GO_x_ bio-HJs) via PDA linkage, targeting sugar-rich cariogenic microenvironments. The charge separation facilitated by MXene-Ag₃PO₄ heterostructures boosted ROS generation efficiency. Furthermore, these heterojunctions exploited GO_x_ to decompose biofilm glucose while generating H₂O₂ as substrates for chemodynamic and photodynamic processes. Under NIR irradiation, MX/AgP-GO_x_ bio-HJs exhibited multiple bactericidal effects through the combination of metal ion therapy, PTT, and enhanced CDT/PDT. This cascade achieved efficient biofilm eradication (log reduction: 1.45 log₁₀ colony-forming units/ml). Crucially, dark-phase antibacterial activity persisted via triple mechanisms: Ag^+^ bactericidal action, Ag^0^ NP catalytic activity, and GO_x_-mediated glucose depletion, overcoming phototherapy’s light dependency limitation.

Current research predominantly focuses on constructing MXene heterojunctions for efficient antibacterial PDT, yet enormous concerns emerge when deploying such coatings on Ti implants. The high-concentration fluorine-based etching essential for MXene synthesis poses substantial toxicological and environmental hazards, necessitating multiple purification steps for the implant surface to eliminate fluorine terminations—a process that increases manufacturing complexity and costs. Moreover, residual fluoride content may induce severe health complications. To address this, researchers have developed an effective alternative approach using fluoride salt/HCl etchant mixtures that circumvent in situ hydrofluoric acid (HF) [139]. Precise control over coating dimensions, dispersion, and thickness remains critical as most of the MXene coatings readily lose their hydrophilicity due to aggregation. Bioinspired MXene coating strategies, such as mussel-inspired PDA modification, have thus been proposed to enhance biocompatibility, leveraging multiple interfacial interactions for robust adhesion and long-term stability [135]. Last but not least, the toxicological profile of MXene-modified implants presents serious biomedical challenges: Excessive release of metal ions or particulates from heterojunction coatings can induce adverse inflammatory responses and cytotoxicity. With only limited studies evaluating the biodistribution of MXene-functionalized implants to date, a comprehensive pharmacological assessment becomes imperative.

##### Metallic elements

Gold nanoparticles (Au NPs) have been extensively utilized in anticancer and antibacterial PTT owing to their unique LSPR phenomenon and superior photothermal characteristics [140]. Tang et al. [4] proposed the application of Au NPs in Ti implant photodynamic treatment by incorporating rare earth nanoparticles (RENPs) and Au NPs onto Ti-supported TiO_2_ nanotube arrays. In this system, RENPs specifically facilitated the upconversion of NIR light to a visible spectrum that could be absorbed by Au NPs, which in turn transformed the photon energy into hot electrons and lattice vibrations. The hot electrons went directly to the contact material or partially interacted with surrounding water and oxygen molecules to produce ROS, while lattice vibrations caused localized temperature elevations. Under NIR light, the synergistic effects of photo-responsive currents from extracellular electron transfer (EET), photothermal, and photodynamic mechanisms greatly impaired the membrane structures and activities of *E. coli* and *S. aureus*, achieving effective antibacterial performance. Following the suppression of infections, Au NPs reinstated the cellular osteogenic potential and collaborated with the nanostructure and the photocurrent to enhance new bone formation. Consequently, the Au NP-assisted implants managed to eradicate bacterial biofilms and modulate the osteogenic process. To comprehensively elucidate the influence of photocurrents on osteogenesis, it is recommended to further explore the potential mechanisms highly associated with bone regeneration, including the mitogen-activated protein kinase (MAPK) signaling pathway, RHO/RHOK signal, and Ca^2+^ influx.

As a distinct morphological variant of Au, gold nanorods can achieve a broad spectrum of light absorption from the visible to NIR windows by modifying their aspect ratio [141]. Wang et al. [142] integrated gold nanorods with Bi_2_S_3_ to create Au@Bi_2_S_3_ Schottky junction nanocomposites. Under 808-nm laser irradiation, photogenerated electrons from Bi_2_S_3_ can swiftly move to the Au nanorods, leading to the high separation efficiency of electron-hole pairs. Furthermore, the urchin-like Au@Bi_2_S_3_ core-shell architecture demonstrated remarkable photothermal conversion efficiency. Consequently, the augmented synergistic effects of PDT and PTT enabled Au@Bi_2_S_3_ to rapidly inactivate bacteria. Similar to Au, Pt is another noble metal whose doping into TiO_2_ enhances visible light absorption and charge separation [143]. Specifically, Pt NPs function as electron sinks, suppressing recombination of photogenerated carriers and thereby amplifying ROS generation [144]. Yang et al. [145] designed a TiO_2_/Pt system with spatially confined Pt NPs within anatase nanotubes by template-assisted ALD. The incorporation of Pt narrowed TiO_2_ bandgap (from 3.30 to 3.05 eV), enhancing visible light-triggered ·OH generation. As a result, this system achieved 100% and 97.38% antibacterial efficacy against *E. coli* and *S. aureus*. Importantly, this TiO₂/Pt nanozyme also possessed robust CAT/SOD-like activities, inducing H_2_O_2_ decomposition into O_2_ to alleviate hypoxia while scavenging O₂·^−^ to mitigate oxidative damage, thereby enabling a coordinated transition between bacterial eradication and tissue healing.

Monometallic nanoparticle coatings may suffer from insufficient photoreactivity and cytotoxicity caused by high-dose ion release [146]. To cope with these issues, some studies have laid their focus on bimetallic nanoparticles, which demonstrate superior photothermal, photocatalytic, and enzyme-like activity over monometallic NPs. In bimetallic systems, heterometallic bonding and lattice distortion generate abundant catalytically active sites, while architecturally complex structures (e.g., hollow, porous, or core-shell configurations) enhance surface plasmon resonance (SPR) effects [147]. By precisely controlling nanoparticle size, morphology, and composition, bimetallic NPs are expected to simultaneously enhance photoreactivity and reduce cytotoxicity through electronic coupling effects between the metallic elements. In Osman et al.’s research [146], hydroxyapatite (HA), Ag–Cu NPs, and poly(pyrrole) (PPy) were integrated onto the Ti implant surface via electrochemical deposition. PPy promoted the in situ reduction of Ag^+^/Cu^2+^ to generate bimetallic NPs while avoiding nanoparticle aggregation. Moreover, it rendered the coating NIR responsiveness to induce high temperatures (>50 °C) and ROS generation under the SPR effect of Ag–Cu bimetallic NPs. This coating with the hierarchical architecture demonstrated multifunctional capabilities: Its Ag–Cu NP surface layer achieved rapid antibacterial action through PTT/PDT synergy; concurrently, the intermediate HA layer released Ca^2+^/PO₄^3−^ in physiological gradients to simulate mineralization, while the underlying PPy layer functioned as an ion-regulating reservoir, enabling sustained release of bioactive Cu^2+^ for cellular behavior modulation.

##### Metal-organic frameworks

Metal–organic frameworks (MOFs), synthesized through coordination of metal ions/clusters with polytopic organic ligands, exhibit ultrahigh porosity, extensive surface areas, unsaturated metal sites, and tunable functionality. These attributes render them versatile platforms for gas storage, electrochemistry, catalysis, photothermal/photodynamic treatment, and other biomedical applications [148]. Their high surface area and flexible structures enable precise incorporation of photosensitizer ligands into crystalline frameworks, effectively mitigating molecular aggregation and self-quenching while enhancing aqueous solubility. A representative example involves porphyrin-based MOFs [e.g., tetracarboxyphenyl porphyrin (TCPP)], where functionalized free porphyrins serve as organic linkers coordinated with metal centers. In such structures, porphyrin-based MOFs have large π-conjugated electron heterocycles and extra lone pair electrons, which not only render them enhanced susceptibility to electron transfer but also allow the selection of metal nodes of MOFs to improve biocompatibility [149]. Li et al. [150] engineered Cu-TCPP@MnO_2_ composites by uniformly depositing MnO_2_ oxygen-generating nanozymes on Cu-TCPP MOF substrates, overcoming limitations in ROS generation during PDT within hypoxic bacterial infection microenvironments. In this design, MnO_2_ catalyzed H₂O₂ decomposition in weakly acidic infected sites to produce O₂, H₂O, and Mn^2+^, thereby supplying essential substrates for PDT. Furthermore, Cu^2+^ and Mn^2+^, whose release was thermally accelerated, could undergo Fenton-like reactions to directly yield ^1^O₂ and ·OH radicals, degrading biofilms and depleting overexpressed GSH, thus further improving the antimicrobial efficiency. Leveraging TCPP’s dual functionality as an agent for both PDT (660 nm) and PTT (808 nm), dual-photo-excitation of Cu-TCPP@MnO_2_ enabled triple synergistic antimicrobial action combining PTT/PDT/CDT, achieving >99.9% inhibition against *S. aureus*.

Porous MOFs can also physically encapsulate small-molecule photosensitizers through interactions like hydrogel bonding, hydrophobic interaction, or π–π stacking. For instance, Yu et al. [151] engineered customized MOFs [MIL-100(Fe)] wherein size-matched IR775 dye molecules were accommodated in “single rooms”, minimizing dye–dye interactions and achieving exceptional photostability. Remarkably, only 2% of the encapsulated dye was released after 10 d at 37 °C. Upon NIR activation, this system achieved 98.5% elimination efficiency against MRSA in vitro, attributed to the combination of low-dose PDT and intrinsic antibacterial properties of IR775. Nevertheless, such noncovalent loading approaches may risk premature photosensitizer leakage and suboptimal drug-loading efficiency. Covalent post-synthetic modification has therefore been developed for improvement [148]. By this way, the photosensitizers are attached to the linkers or frameworks via covalent coupling, resulting in optimized loading efficiency and stability. Besides, metal ions (e.g., Fe^2+^ and Ag^+^) released during MOF degradation further potentiate antibacterial activity through direct bactericidal effects or Fenton reactions [148]. The bioactive ions (e.g., Mg^2+^ and Zn^2+^) liberated from frameworks like Zn/Mg-MOF-74, ZIF-8, and ZIF-67 can also promote osteogenesis and angiogenesis, thereby accelerating the osseointegration of Ti implants [152].

Collectively, both photosensitizer-loaded and intrinsically photosensitizing MOFs can achieve superior antibacterial outcomes through PDT, PTT, antimicrobial drug/ion release, and their combinations. Deploying tailored MOF-based coatings on Ti implants as antibacterial interfaces holds great promise for combating infections and enhancing osseointegration. Nonetheless, a critical challenge persists: Most organic linkers in current MOF designs lack clinical approval [152]. Given the gradual decoupling of coordinated metal species and organic linkers in biological environments, the inevitable release of these linkers poses serious health risks that cannot be overlooked. Consequently, when engineering MOF-modified Ti implants, strategic selection of metal centers and optimization of MOF structural stability become imperative.

#### Alternative inorganic photosensitizers

Phosphorus is an essential element in the human body, comprising roughly 1% of total body weight. Black phosphorus (BP), a subject of advanced research in 2D semiconductors, features a layered nanostructure, a high specific surface area, and a broad tunable bandgap range (0.3 to 2.0 eV). The distinctive characteristics of BP nanosheets, including elevated carrier mobility, outstanding biocompatibility, and high photothermal conversion efficiency, render them highly suitable for photothermal and photodynamic therapy. [153]. Meanwhile, BP, as a phosphorus source, can naturally degrade to yield PO_4_^3−^, with the oxidation process accelerating the release rate. Its excellent in situ biomineralization capacity is conducive to osteogenesis and bone regeneration [154]. Nevertheless, the atomic layers of BP will deteriorate swiftly when subjected to environmental conditions, thus limiting its practical application. The modification of BP for enhanced physiological stability has been investigated by many studies, such as polyethylene glycol-amine (PEG-NH_2_)-treated BP, GO-coated BP, and PDA-modified BP [154]. To address tumor recurrence, postoperative bacterial infection, and bone defects in patients with bone cancer, Li et al. [155] formulated a multifunctional GelMA/dopamine methacrylate hydrogel coating incorporating 2D BP nanoparticles safeguarded by PDA (pBP) on the surface of poly(phthalazinone ether nitrile ketone) (PPENK) implants through a photocrosslinking technique. The integration of PDA markedly increased the stability of BP and improved its photothermal efficacy. Under 808-nm laser irradiation, the pBP could regulate the release of doxorubicin hydrochloride through temperature elevation while simultaneously generating ROS through its photodynamic effect, thereby effectively eradicating bacterial infections. During the gradual disintegration process, pBP efficiently eliminated excess ROS, averting ROS-induced damage to healthy cells. Moreover, its breakdown product, PO_4_^3−^ with exceptional mineralization capacity, actively drove osteogenesis, hence promoting the osseointegration of PPENK implants even in the presence of bacterial circumstances.

RP, an allotrope of phosphorus, has been documented as a photothermal coating material for Ti implants [69,156]. In comparison to BP, RP has favorable biocompatibility while being more economical. A work by Mao et al. [157] created a P–N heterojunction comprising oxide perovskite-type calcium titanate (CTO) and fibrous RP on the surface of Ti implants. Upon close contact between the 2 semiconductors, the RP and CTO attained a uniform Fermi energy level, resulting in a displacement of the relative positions of the VB and CB, with the edge potentials of the VB and CB of the RP elevated by 1.71 eV, while those of the CTO were depressed by 0.64 eV. Consequently, the CB position of the CTO shifted to −0.54 eV, which is more negative than that of O_2_//O_2_^•−^ (−0.33 eV), thereby facilitating the generation of ROS. The CB of the RP was positioned higher than that of the CTO, creating a band offset that facilitated the separation and transfer of photoinduced electrons from the CB of the RP to the CB of the CTO. Therefore, the combination of the internal electric field and band offset successfully separated and transported electron-hole pairs stimulated by NIR light, greatly enhancing photodynamic ROS yields. The charge transfer also imparted exceptional photothermal characteristics to the Ti-CTO/RP heterostructure. Under NIR irradiation, the synergistic impact of PDT and PTT resulted in the swift elimination of mature MRSA biofilms, demonstrating an in vivo effectiveness of 99.42%. Additionally, the CTO/RP nanofilm served as a calcium and phosphorus supply, offering an osteoconductive platform that facilitated the proliferation and osteogenic differentiation of BMSCs, hence promoting bone regeneration and osseointegration following the eradication of infection (Fig. 9E to G). The application of BP and RP exhibits immense potential for sequentially eliminating infection and promoting bone integration, warranting sufficient future attention.

Collectively, a broad spectrum of organic and inorganic photosensitizers has been engineered for Ti implants. Their advantages and disadvantages have been listed in Table 2. Given the hypoxic microenvironment characteristic of bacterial biofilms, oxygen-independent type I photosensitizers may offer superior efficacy. Concurrently, various strategies to augment oxygen levels for potentiating type II photosensitizers have been exploited, which will be discussed in Therapeutic enhancement via oxygen supply. In current research, almost all organic photosensitizers and certain inorganic photosensitizers are immobilized onto Ti implants via surface coatings. Diverse coating techniques, particularly the widely adopted versatile mussel-inspired PDA-based coating, provide a facile, rapid, and cost-effective approach for photosensitizer immobilization. Nevertheless, such coatings face inherent risks of delamination and compromised durability under mechanical stress during surgical implantation. In contrast, numerous inorganic photosensitizers (notably modified TiO₂) can be directly synthesized in situ on Ti substrates through hydrothermal methods, circumventing coating-related limitations. However, this technique demands precise control over critical parameters (e.g., temperature, pressure, pH, and reaction duration) and incurs higher costs compared to conventional coating techniques. Compounding this issue, current research disproportionately prioritizes functional enhancement of modified implants while marginalizing critical translational determinants—particularly fabrication scalability, long-term stability in physiological environments, and cost-effectiveness. Crucially, rigorous consideration of these factors is imperative for advancing clinical translation.

**Table 2.** The advantages and disadvantages of common photosensitizer materials

Common photosensitizer materials	Advantages	Disadvantages
Methylene blue [58,223]	FDA-approved photosensitizer, relatively high safety, hydrophilicity, positively charged, easily passes through Gram-positive and Gram-negative bacterial membranes	Cationic dye-associated health problems, rapid enzymatic reduction in the biological environment
Indocyanine green [63,64,68]	FDA-approved photosensitizer, relatively high safety, strong light absorption in the NIR region, fluorescent properties, photodynamic/photothermal properties	Instability, photobleaching properties, photodegradability, thermal degradation, rapid clearance
Curcumin [70,224]	Relatively high safety, inherent anti-inflammatory, antioxidant, and antimicrobial properties, minimal dark toxicity, high photo-activity rate	High hydrophobicity, limited chemical stability, only activated by blue light
Chlorin e6 [77,78]	FDA-approved photosensitizer, relatively high safety, inherent anti-inflammatory properties, large absorption coefficient, low residue in the body	Tendency to aggregate, poor water solubility, easy removal
TiO_2_ [80,143]	FDA-approved implants, high biocompatibility, high photostability, in situ construction on titanium implants, tailored nanostructure, diverse modification	Only activated by UV, high recombination rate of photogenerated charge carriers, requiring additional modification
Carbon-based nanomaterials [110,120,225]	Biocompatibility, high photostability, good water dispersion, broad absorption, photodynamic/photothermal properties, inherent antibacterial activities, ease of modification	Potential long-term toxicity, insufficient photosensitizing activity
Metallic-based photosensitizers [26,124]	High photostability, helpful effects of metal ions, broad absorption, photodynamic/photothermal properties, inherent antibacterial activities, diverse kinds	Hard to degrade, potential cytotoxicity by metal ion accumulation, relatively complex processing
Black/red phosphorus [154–156]	High biocompatibility, tunable band gap, extensive NIR absorbance, photodynamic/photothermal properties, degrading into PO_4_^3−^ conducive to mineralization	Instability, relatively high cost of BP

## Challenges and Perspectives

The rise of super-resistant bacteria has markedly diminished the efficacy of antibiotics, and bacterial biofilm infections pose a substantial risk to the durability of Ti implants and bone integration. Consequently, the advancement of novel antibacterial methods is essential. As previously mentioned, PDT possesses remarkable potential for eliminating bacteria and biofilms; nevertheless, both the organic and inorganic photosensitizers presently employed have limitations. Before the future application of PDT in the antibacterial treatment of Ti implants and its translation to clinical practice, numerous problems must be resolved, including but not limited to the following [22,28]:

· The inefficacy of antimicrobial action: Based on the principles of photodynamic reactions, the hypoxic microenvironment of biofilm infections and inadequate blood supply at implant sites frequently result in diminished oxygen levels, leading to an insufficient quantum yield of ^1^O₂ in type II PDT. The high recombination rate of photogenerated electron-hole pairs severely hampers ROS production efficiency in type I PDT. Furthermore, PDT alone often does not yield satisfactory therapeutic results.
· The limited tissue penetration of exciting lights: Most organic and certain inorganic photosensitizers predominantly absorb lights with shorter wavelength, which inadequately penetrate tissue, hence restricting their application in the treatment of biofilm infections on Ti implants.
· The intrinsic shortcomings of photosensitizers: Their comparatively feeble light absorption capacity culminates in suboptimal light consumption, resulting in energy wastage. Organic photosensitizers frequently encounter issues with physicochemical stability and photostability, whereas biocompatibility is a major limitation for inorganic photosensitizers.
· Challenges in bone integration: The primary objective of infection management is to establish an optimal environment for osseointegration between Ti implants and host bone tissue. Nonetheless, the majority of current photosensitizers solely demonstrate the capacity to produce ROS upon light activation, devoid of advantageous bioactivities such as immunomodulation, angiogenesis, and osteogenesis that are vital for osseointegration. Consequently, additional modification of Ti implants is frequently necessary to enhance osteoconductivity and biocompatibility.

In light of the present difficulties encountered by photodynamic treatment in Ti implants, we offer and examine several viable scientific solutions grounded in the available literature.

### Optimization of PDT systems

Photosensitizer stability is vital to guarantee therapeutic efficacy in PDT. This necessitates stabilizing heavy metal-containing photosensitizers in vivo to avert toxic ion leakage and developing effective loading methods for organic photosensitizers on Ti implants to prevent their rapid deterioration in biological settings. Concerning this issue, various innovative methods, including the fabrication of core-shell structures and the utilization of highly biocompatible materials such as PDA, MPDA, PEG, mesoporous silica nanoparticles (MSNs), and liposomes, have been devised to enhance the stability and biocompatibility of photosensitizers [17]. Compared with free photosensitizers, photosensitizers loaded by these nanocarriers possess the following advantages: (a) improved water solubility, (b) tunable surface potential and facile modification of targeted molecules, (c) selectivity of bacteria over ambient mammalian cells, and (d) preventing free photosensitizers from invalid dimerization or trimerization [158].

PDA and MPDA are the most studied and have gained widespread adoption. PDA, a biopolymer inspired by mussels, demonstrates universal adhesion, enduring stability, exceptional biocompatibility, and photothermal conversion capacity. Moreover, the distinctive chemical structure of PDA, which includes several functional groups such as catechols, amines, and imines, facilitates covalent bonding with organic molecules and the adsorption of metal ions [159]. Multifunctional PDA coatings have been extensively utilized on Ti implants, demonstrating great promise in the immobilization of organic photosensitizers and the improvement of the biocompatibility of inorganic photosensitizers. MSNs are distinguished by their unique properties, such as tunable porous structures (2 to 50 nm), large surface area, high loading capacity, facile surface functionalization, and excellent biocompatibility, making them highly regarded as promising nanocarriers for antibacterial agents [160]. In the context of PDT, MSNs are identified as a potential nanoplatform for photosensitizers owing to their optical transparency to light absorption [161]. Moreover, MSNs can realize bacterial targeting by the strategic design of MSN surface charge and the functional group grafting [160]. Various engineering strategies also endow MSN composites with stimulus responsiveness. Zhang et al. [162] prepared MSNs as a substrate for the in situ deposition of mPL NPs (persistent luminescence NDs of ZnGa_2_O_4_:Cr^3+^), followed by surface coupling with silicon phthalocyanine (Si-Pc) and the electrostatic assembly of Cy7. The resultant composite, designated mPL@Pc-Cy NPs, exhibited pH-responsive behavior, enabling spontaneous fluorescence activation in response to the acidic microenvironment of bacterial infections for real-time tracking. Simultaneously, mPL protonation under acidic conditions induced electrostatic repulsion, resulting in Cy7 disassembly and the suspension of the fluorescence resonance energy transfer (FRET) effect. Utilizing mPL NPs as an internal light source to continuously excite Cy7, this smart platform overcame the short durability of traditional PDT. The amalgamation of photosensitizers with MSNs has yielded satisfactory results: It not only reduces cytotoxicity and enhances biocompatibility but also augments solubility and stability in a physiological environment, remarkably enhancing the performance of hydrophobic and less stable photosensitizers [161].

Light is another basic element in the PDT system, and the selection of an appropriate light source is closely associated with PDT efficacy. The type, wavelength, power intensity, and duration of light exposure profoundly impact the antibacterial outcomes. Light sources utilized in dental and orthopedic fields for PDT predominantly consist of 2 types: LEDs and lasers [163]. LEDs can emit light either in the visible region or in the NIR region (>700 nm). Owing to its cost-effectiveness, user-friendliness, and widespread acceptance, LED is a frequently utilized light source in dentistry. Nonetheless, inadequate tissue penetration and diminished intensity limit its efficacy in PDT [164]. Lasers are highly directed and monochromatic, exhibiting exceptional coherence and high intensity, rendering them suitable light sources that may be precisely manipulated to correspond to a specific photosensitizer [163]. With their intensified and focused energy beams, lasers are particularly effective in eradicating deeper site infections. However, if not executed correctly, such as by establishing excessive power intensity or irradiation duration, lasers may result in overheating and damage to the surrounding tissue. Conversely, LED light is characterized by low intensity, lacking directionality and coherence, resulting in enhanced safety due to less heat production [164]. Moreover, lasers generally require expensive special equipment and regular maintenance, rendering them less accessible than LEDs in therapeutic settings. Nowadays, the concept of X-PDT utilizing x-rays as an excitation source has been advocated by numerous studies for cancer treatment [165,166]. X-rays, a kind of electromagnetic radiation, exhibit virtually unlimited penetration capabilities in biological tissues and bones. Nonetheless, classified as a “carcinogen” by the World Health Organization, these high-energy particles inevitably have adverse consequences on the adjacent normal tissues during treatment. Maintaining high therapeutic efficiency while minimizing radiation doses remains a tremendous obstacle [166]. In conclusion, when administering PDT in the human body, it is imperative to meticulously evaluate the precise parameters of the excitation light and achieve an appropriate equilibrium between safety and efficacy.

The excitation light wavelength also makes a great difference to PDT effectiveness. Typically, the depth of light penetration in tissue is highly correlated with its wavelength. The penetration depth is found to be 5 to 6 mm at 700 to 800 nm, 2 to 3 mm at 630 nm, 1 mm at 500 nm, and <0.5 mm at 400 to 430 nm [164]. Owing to the restricted penetration depth (<3 mm), higher dosages may be necessary to maintain the therapeutic efficacy of visible light-excited PDT for deeper tissues, hence increasing the risks of skin burns or photosensitivity. Longer-wavelength light (>800 nm) is safer due to its comparatively reduced energy transfer and ability to penetrate deeper into the skin. Therefore, recent studies on Ti implant infections have focused on the light wavelengths in the NIR region, encompassing NIR-I (700 to 900 nm) and NIR-II light (1,000 to 1,700 nm). NIR-I light still encounters constraints in reaching deeper implant locations due to considerable dispersion and absorption by tissues. In contrast, NIR-II light presents advantages such as reduced energy dissipation, enhanced tissue penetration, elevated skin tolerance, and lower toxicity, rendering it more appropriate for addressing implant-associated infections [84,167]. Consequently, the advancement of NIR-II photosensitizers for Ti implants represents a viable approach.

### Aggregation-induced emission-active photosensitizers

**Fig. 10.**
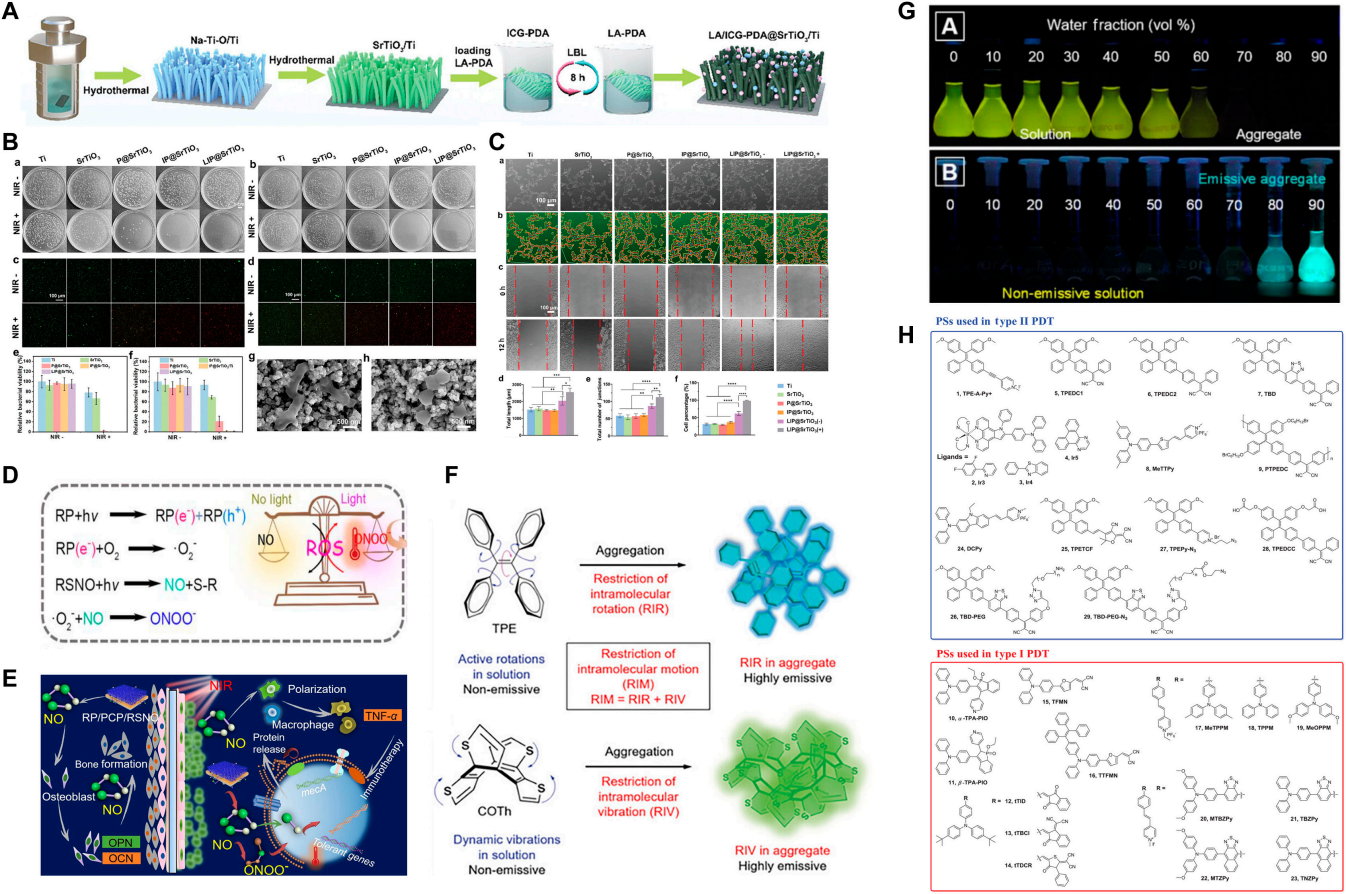
(A) Schematic diagram of the synthesis of LIP@SrTiO_3_ on the Ti substrate. (B) In vitro antibacterial ability of LIP@SrTiO_3_-Ti. (C) Vascular regenerative capacity of LIP@SrTiO3. Reproduced with permission [192]. Copyright 2024, Wiley. (D) Mechanism of Ti-RP/PCP/RSNO to generate NO and ·ONOO^−^. (E) Schematic diagram of Ti-RP/PCP/RSNO promoting bone formation through M1 polarization of macrophages while eradicating MRSA biofilm through gene down-regulation. Reproduced with permission [194]. Copyright 2020, ACS Publications. (F) Schematic illustration of the RIM mechanism by combining the restriction of intramolecular rotations (RIR) and restriction of intramolecular vibration (RIV) concepts in AIEgens. Reproduced with permission [220]. Copyright 2021, Elsevier. (G) Aggregation-caused quenching (ACQ) and aggregation-induced emission (AIE) phenomenon. Reproduced with permission [221]. Copyright 2022, MDPI. (H) Molecular structure of some AIE photosensitizers. Reproduced with permission [222]. Copyright 2021, Wiley.

Since the hematoporphyrin derivative (HpD) was identified as the first photosensitizer in the 1960s, numerous organic photosensitizers have been developed and approved by the U.S. FDA for clinical applications. However, many conventional photosensitizers suffer from aggregation-caused quenching (ACQ) due to their planar architectures and overall stacking interaction, which diminishes fluorescence emission and ROS generation, severely compromising PDT efficacy [168]. Aggregation-induced emission luminogens (AIEgens), first reported by Tang and colleagues [169] in 2001, counteract ACQ by suppressing nonradiative decay in aggregated states, exhibiting dramatically enhanced luminescence in solid or concentrated environments. Their twisted molecular conformations restrict intramolecular motion, conferring not only tunable emission wavelengths, large Stokes shifts, and exceptional photostability but also augmented ROS generation through aggregation-enhanced ISC, establishing AIEgens as the ideal candidates of photosensitizers in PDT (Fig. 10F to H) [168]. Concurrently, nonradiative energy dissipation synergistically facilitates photothermal effects, enabling efficient PTT. While exhibiting minimal fluorescence in dilute solutions, AIEgens can elicit intense emission of NIR fluorescence by accumulating in the cytoplasmic membrane of pathogens, making them an outstanding integrated multimodal theranostic platform for combating infections [170].

The molecular design of AIE-active photosensitizers critically determines their PDT efficiency, thus attracting substantial attention. Established optimization strategies include heavy-atom incorporation into molecular skeletons, donor–acceptor architecture construction, cationization engineering, and polymerization approaches [171]. Nevertheless, most AIEgens exhibit solubility limited to organic solvents, which restricts their penetration through bacterial membranes, resulting in inadequate antibacterial performance that precludes clinical applications. To address these limitations, several methods have been developed [172]. Covalently conjugating ionic or hydrophilic chains to AIEgens can yield water-soluble AIEgen analogs or amphiphilic AIEgen macromolecules that readily self-assemble into AIEgen nanoparticles, thereby enhancing their hydrophilicity. Encapsulation strategy using amphiphilic copolymers [e.g., 1,2-distearoyl-sn-glycero-3-phosphoethanolamine (DSPE)-PEG-wrapped hydrophobic AIEgens] also proves effective and can achieve precise control of their size. Alternative nanoplatforms, including BSA, MSN, cell-membrane capsules, and liposomes, have also been exploited for AIEgens’ delivery to infected regions [170]. Furthermore, integrated therapeutic approaches combining PDT, PTT, gas therapy, and immunotherapy have been developed to synergistically augment treatment outcomes [171].

Despite AIE-active photosensitizers emerging as a focal point in phototheranostics for cancer and infection [173], their utilization in orthopedic implants remains underexplored, with only a few investigations. Wang et al. [174] grafted methyl-PEG4-alcohol onto both hydroxyl groups of curcumin, followed by modification of its diketone moiety to create a BODIPY-like architecture. This yielded a red-emissive AIEgen (4BC) exhibiting efficient ROS generation. Subsequently, the 3D-printed 4BC@TMP scaffold was fabricated via precipitation–adsorption methodology. Under UV irradiation, the implanted scaffold demonstrated excellent in situ imaging capability coupled with potent bactericidal activity against *E. coli* and *S. aureus* through PDT. In a separate study, Wang et al. [175] employed a biomimetic nanomedicine strategy to load AIEgens within bacterium-like particles derived from *Lactobacillus fermentum* probiotic strains, which exhibited minimal immunogenicity and were further immobilized on polyoxazoline coatings. The molecular framework of the AIEgen consists of α-cyanostilbene with strong electron-donating groups (triphenylamine) and strong electron-accepting groups (pyridine). Due to restricted intramolecular motion (RIM), its propeller-like structure achieved excellent emission in aggregated states. Additionally, the positive charge of the pyridine moiety can attract negatively charged viruses and bacteria. Under light irradiation, these coatings utilized ROS to selectively kill *S. aureus* and *Pseudomonas aeruginosa* without harming normal cells. Concurrently, inflammatory responses were suppressed, thereby reducing the possibility of implant rejection.

In summary, AIE-active photosensitizers with dual antibacterial/imaging functionality demonstrate promising potential for controlling Ti implant-associated infections. Future research should prioritize the development and application of NIR-absorbing/emitting AIEgens with superior tissue penetration capacity and enhanced biocompatibility [176].

### Therapeutic enhancement via oxygen supply

In type II PDT, the therapeutic efficacy is critically dependent on ambient oxygen levels, which directly determine the yield of ^1^O_2_ [177]. However, the barrier properties of bacterial biofilms impede oxygen availability, resulting in hypoxic microenvironments at infection sites that severely diminish the oxygen-dependent bactericidal efficacy of type II PDT [51]. Moreover, the photochemical reaction central to PDT itself rapidly consumes local oxygen, exacerbating hypoxia. Extensive research has focused on strategies to improve oxygen availability via enhanced oxygen delivery or in situ oxygen generation.

Natural and artificial oxygen carriers demonstrate promising potential for oxygen transport to hypoxic regions, thereby augmenting ^1^O_2_ generation. Hemoglobin, the principal oxygen transport protein produced by erythrocytes in mammalian circulatory systems, exhibits reversible oxygen-binding capacity. Hemoglobin-based oxygen vehicles have been widely exploited via covalent conjugation or encapsulation techniques to facilitate oxygen delivery in hypoxic environments [178]. Erythrocytes, as natural hemoglobin reservoirs, contain approximately 270 million hemoglobin molecules per cell, with each hemoglobin capable of binding up to 4 O_2_ molecules. This characteristic makes biocompatible erythrocyte–photosensitizer conjugates appealing for improving oxygen delivery [179]. Wan et al. [180] engineered nanoscaled erythrocytes containing oxyhemoglobin and gas-generating agents for the co-loading and controlled release of ICG. This combination exhibited notably improved PDT efficiency compared to free ICG. Perfluorohexane (PFH), an established oxygen carrier sanctioned by the U.S. FDA, exhibits a remarkable capacity for oxygen storage, maintaining oxygen levels 20 times higher than those of water at equivalent oxygen partial pressure. Wang et al. [181] incorporated PFH and IR780 into the hydrophobic core of liposomes, creating an improved NIR-excited PDT system [Lip(IR780 + PFH)] on Ti-based implant surfaces. After 15 min of 808-nm NIR irradiation, Lip(IR780 + PFH) exhibited remarkable antibacterial efficacy of 99.62% against *E. coli* and 99.63% against *S. aureus*. Contrastingly, the PDT system without PFH modification exhibited markedly diminished antibacterial activity, with efficiencies of merely 66.54% and 48.04%, respectively. Besides, polymeric materials such as MOFs and covalent organic polymers/frameworks can serve as an ideal platform for oxygen storage and delivery, owing to their porous architectures and substantial surface areas [182]. For example, zirconium (IV)-based MOF (UiO-66) was employed to capture and transport molecular oxygen via physical adsorption, hence enabling efficient oxygen delivery to hypoxic regions during the ICG-mediated PDT [183]. Nevertheless, challenges persist when deploying these oxygen carriers on Ti implants, primarily concerning loading/structure instability and oxygen premature leakage.

In situ oxygen generation strategies harnessing chemical reactions to produce oxygen have been extensively explored to address hypoxia-related limitations. In the acidic microenvironment characterized by elevated levels of H_2_O_2_ around tumors or bacterial biofilms, the most commonly employed approach is to conduct the reaction between H_2_O_2_ and MnO_2_ [184]. In the study by Jiang et al. [53], MnO_2_ was incorporated into the design of PDT nanoparticles. MnO_2_ facilitated the breakdown of ambient H_2_O_2_, hence enabling efficient oxygen generation to improve PDT efficacy. Additionally, the released manganese ions acted as immunostimulatory agents, synergistically enhancing adaptive immune responses to bacterial infections. Besides, artificial CAT mimetics including Pt NPs, CeO_2_, Prussian blue, and iron-based nanocatalysts, as well as the natural enzymes like CAT, have been documented to facilitate the decomposition of H_2_O_2_ for hypoxia alleviation and PDT enhancement [182,185]. These biocatalysts are susceptible to degradation and clearance in intricate physiological environments, requiring a steady drug delivery system to shield them from premature degradation.

The availability of endogenous H_2_O_2_ may occasionally be restricted; thus, alternative oxygen sources have been explored. CaO_2_, a highly biocompatible metal peroxide, can quickly disintegrate in aqueous solutions to generate H_2_O_2_ and O_2_ concurrently, thereby enhancing the efficiency of PDT [186]. Inspired by the natural phenomenon that chloroplasts in green plants efficiently catalyze the synthesis of oxygen from water under light irradiation, researchers have developed PDT systems that incorporate water-splitting nanoparticles for photodecomposition of physically abundant water, thereby circumventing the constraint of insufficient endogenous H_2_O_2_. C_3_N_4_-based nanoparticles and graphdiyne oxide nanosheets have been engineered as advanced materials for effective photocatalytic water splitting, whose application in PDT merits further investigation [120,187]. Recently, photosynthetic microorganisms, particularly cyanobacteria that perform oxygen-evolving photosynthesis, have emerged as promising candidates for oxygen self-supportive PDT due to their high biosafety, controllable photoautotrophy, low cost, and excellent oxygen yield [188,189]. Nonetheless, the introduction of these living organisms onto bionert Ti and the assurance of their functionality in vivo present a huge challenge.

### Synergistic antibacterial approaches

Despite its antibacterial promise, PDT’s practical implementation faces multifactorial limitations. Beyond documented challenges with light sources and photosensitizers, its principal antimicrobial effector, ROS, has an inherent short half-life and limited tissue penetration. Treating severe infections therefore usually requires high ROS fluxes, increasing the risk of off-target oxidative damage to healthy cells. Since standalone PDT often delivers suboptimal clinical outcomes, strategic integration with complementary therapies (e.g., PTT, gas therapy, and SDT) is imperative to unlock its full therapeutic potential.

PTT is regarded as a potential antibacterial approach owing to its multiple advantages, such as noninvasiveness, robust tissue penetration, minimal resistance, and high antimicrobial efficiency. This photothermal sterilization technique entails photothermal agents transforming light energy into heat via photon-induced lattice vibrations when activated by NIR light. The resultant localized elevated temperature not only breaches bacterial membranes, resulting in the release of cellular contents, but also irreparably damages bacterial proteins or enzymes, impairing their physiological processes, finally culminating in bacterial mortality [190]. Nevertheless, pure PTT predominantly necessitates high temperatures over 70 °C to eliminate bacterial biofilms, which inevitably inflict harm on adjacent healthy tissues. Consequently, in vivo PTT is restricted to moderate photothermal temperatures (<50 °C), thereby constraining its broad applicability [66,190]. The integration of PTT with PDT has emerged as a prevalent strategy for addressing bacterial infections in Ti implants. The heat produced by PTT enhances bacterial membrane permeability, allowing ROS to penetrate bacteria more easily. Furthermore, PTT diminishes cellular endogenous antioxidants, such as GSH, hence compromising the bacterial oxidative defense mechanism and thereby amplifying ROS damage. Concurrently, ROS augments the permeability and thermosensitivity of compromised bacterial membranes, synergistically enhancing antibacterial efficacy [70,86,191]. Thus, integrating PTT with PDT lowers the requisite temperature and ROS thresholds for each therapy, minimizing damage to healthy tissues while augmenting antibacterial efficacy.

Recent studies document the integration of nitric oxide (NO) gas therapy with Ti implants. As one of the most extensively studied endogenous gaseous molecules, NO exerts concentration-dependent physiological effects. Low concentrations of NO (1 to 400 nM) confer protection against cellular apoptosis and tissue damage while promoting angiogenesis. At higher concentrations (>1 μM), NO covalently binds bacterial DNA, proteins, and lipids, inducing DNA damage and membrane disruption to function as a broad-spectrum antimicrobial agent [192,193]. Liu et al. [192] employed the concentration-dependent effects of NO to develop an antibiofilm nanoplatform utilizing l-arginine (LA) as NO donors and novel ICG (NICG) as photosensitizers, which were affixed to strontium Ti oxide (SrTiO_3_) nanoarrays on a Ti substrate via a PDA intermediary layer. Under high power density NIR irradiation (1.04 W /cm^2^), NICG was stimulated to produce a substantial amount of ROS, part of which interacted with LA to release a high concentration of NO, which subsequently synergized with ROS and the photothermal heat to eradicate bacterial biofilm. Thereafter, by reducing the NIR irradiation intensity to 0.55 W/cm^2^, low concentrations of NO were produced to facilitate the rebuilding of blood vessels. Consequently, the nanoplatform designed to regulate NO release via the NIR switch accomplished programmed biofilm eradication and revascularization (Fig. 10A to C). Employing ROS-sensitive LA as an NO generator, this innovative investigation fabricated a dual-functional implant with enhanced antibiofilm activity and angiogenesis–osteogenesis coupling, which broadens the application of NO gas. In addition to LA, a separate investigation conducted by Li et al. [194] exploited heat-sensitive S-nitrosuccinic acids (RSNO) for NO production. Specifically, the NIR light-responsive hydrogel (RP/PCP/RSNO) coated on the Ti implant could selectively release NO and O₂·^−^ or solely NO. Upon NIR light stimulation, the photothermal properties of PDA and RP facilitated the swift release of NO with high concentration, which subsequently interacted with O₂·^−^ produced by RP nanofilms, leading to the formation of highly toxic peroxynitrite (·ONOO^−^). The findings indicated that >93.1% of MRSA biofilms were eliminated due to the synergistic effects of ·ONOO^−^, heat, and O₂·^−^ under NIR irradiation, together with the inflammatory M1 polarization facilitated by NO. In the absence of NIR irradiation, slow-release NO enhanced the expression of osteogenic genes and resulted in elevated ALP expression, therefore enhancing the osteogenic potential of Ti implants (Fig. 10D and E). Despite strategies to initiate and modulate NO release, contemporary systems exhibit irreversible and imprecise release kinetics—typically an initial burst followed by gradual depletion. Spatiotemporally controlled NO release remains imperative for minimizing NO-induced cytotoxic and proinflammatory consequences. Alternative NO donors, such as diazonium diolates, N-nitrosamines, metal-nitrosyl complexes, and nitrates/nitrites, also warrant a comprehensive exploration into their therapeutic potential [193]. Moreover, tiny gaseous molecules such as carbon monoxide (CO) and hydrogen sulfide (H₂S) have been investigated for their efficacy in addressing bacterial infections [195,196], highlighting the extensive potential of gas treatment.

SDT, which integrates ultrasound with sonosensitizers, has been extensively studied for cancer and infection treatment. The primary mechanism for SDT-mediated bacterial eradication and biofilm disruption is ultrasound-induced ROS generation, arising from the cavitation and sonoluminescence effects. The former is a rapid physical event triggered by sonosensitizers under ultrasound irradiation, involving the development, expansion, and collapse of cavitation microbubbles. This process releases energy as localized high heat and pressure, initiating pyrolysis of water or sonosensitizers to produce ROS (·OH, O₂·^−^, and ^1^O₂). Concurrently, bubble collapse induces sonoluminescence, which energizes the electron orbitals of the sonosensitizer, leading to the formation of electron-hole pairs that contribute to ROS generation [197]. These sonochemical interactions underpinning SDT provides distinct benefits in microbial suppression, such as robust tissue penetration, minimum invasiveness, and high efficacy against drug-resistant biofilms. Notably, TiO_2_ may serve as effective sonosensitizers due to its intrinsic sonosensitive properties. Su et al. [198] introduced an expedited photo-sonotherapy technique by inducing oxygen deficiency in a Ti implant via sulfur (S)-doping (Ti-S-TiO₂_−*x*_). Without external antibacterial coatings, this system attained an exceptional antibacterial efficiency of 99.995% against *S. aureus* within 15 min of NIR and ultrasound treatments, while NIR treatment alone demonstrated merely 51.033% antibacterial efficiency, underscoring the potent synergy of sonodynamic and photothermal therapy. Therefore, integrated phototherapy–sonotherapy multimodal antimicrobial therapy presents an emerging frontier.

Conventional antibiotic therapy carries inherent risks of bacterial resistance and cytotoxicity due to excessive or high-dose administration [199], while prolonged light exposure in phototherapy may cause collateral tissue damage. To address these challenges, recent studies have proposed a win–win phototherapy–antibiotic strategy that enhances antibacterial efficacy while minimizing associated side effects. This approach employs light-controlled intelligent antibiotic-release coatings to optimize spatiotemporally precise drug delivery. In an investigation by Yang et al. [200], a TiO₂/graphene hybrid metastructure with strong NIR light absorption was fabricated on a medical-grade Ti surface via hydrothermal treatment combined with PECVD. Leveraging graphene’s high drug-loading efficiency mediated by π–π stacking interactions, the coating could efficiently load doxycycline (DOX) and achieve its controlled release via NIR-triggered disruption of π–π stacking. Through the combination of PDT and photothermally enhanced DOX chemotherapy, this system exhibited potent bacterial eradication even at ultra-low antibiotic concentrations and brief NIR light exposure, thereby minimizing cytotoxic side effects associated with high drug concentrations or prolonged irradiation. Crucially, given the well-established clinical safety and efficacy profile of antibiotics, this combined strategy of low-dose antibiotics coupled with mild phototherapy offers enhanced translational potential for clinical Ti implant applications.

### Development of dual-functional implants

Clinical follow-up surveys indicate that roughly 12.8% of unsuccessful total knee arthroplasties in the United States result from aseptic loosening, attributed to inadequate interfacial integration between the implant and healthy bone tissue [3]. The attainment of efficient osseointegration is a paramount determinant for implant longevity, constituting the foremost consideration in orthopedic Ti implant design [104]. However, once bacterial infection occurs, it renders the osseointegration process intractable. At the implant interface, competitive adhesion dynamics between bacteria and osteogenic cells critically determine osseointegration success rates. This mutually inhibitory competition governs 2 distinct outcomes: early-stage infection dominated by bacterial colonization versus long-term osseointegration mediated by osteoblastic activity. Following colonization, biofilm formation further triggers proinflammatory cascades that disrupt the physiological osteogenic microenvironment, ultimately compromising osseointegration efficacy [5]. Consequently, dual-functional implants integrating antibacterial/antibiofilm PDT with osteogenesis-promoting capabilities have emerged as a strategic focus to achieve the ultimate objective of stable osseointegration.

**Fig. 11.**
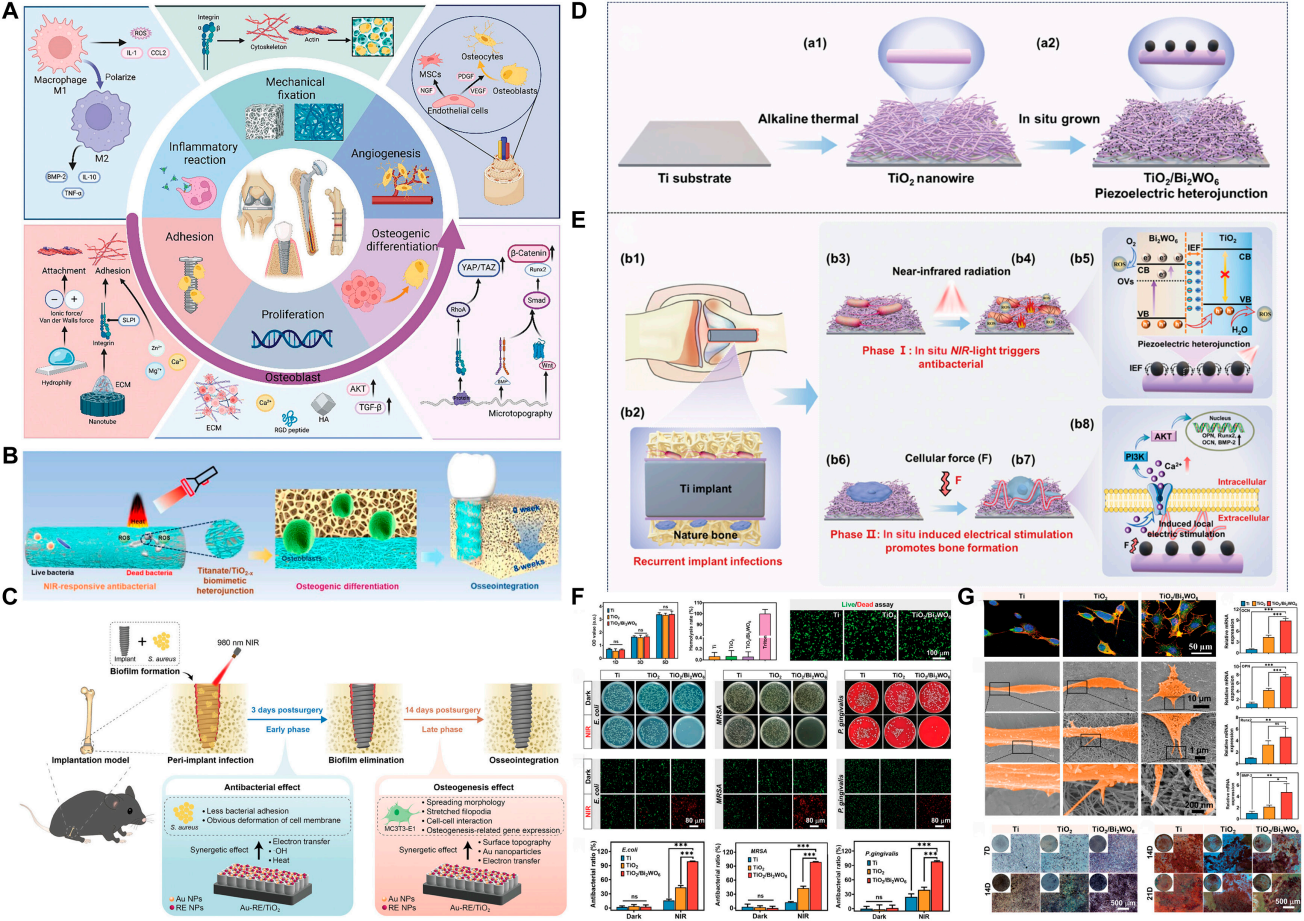
(A) Interface engineering strategy in implant osseointegration. Reproduced with permission [104]. Copyright 2024, Wiley. (B) Schematic illustration of the preparation and dual effects of biomimetic micro/nano-titanate/TiO_2−*x*_ heterostructures on a Ti implant. Reproduced with permission [90]. Copyright 2024, ACS Publications. (C) Schematic diagrams of the dual functions of Au-RE/TiO2 in orthopedic implant infections. The surface topography, Au NPs, and photoinduced electron transfer (photoelectrons) result in a synergetic osteogenic effect. Reproduced with permission [4]. Copyright 2023, Wiley. (D) Schematic illustration of the in situ construction of TiO_2_/Bi_2_WO_6_ on the titanium implant surface. (E) Schematic illustration of the mechanisms of the piezoelectric heterojunctions: NIR light-triggered ROS [facilitated by the built-in electric field (IEF)] and heat production for photodynamic and photothermal antibacterial therapy, and cellular force-induced electrical stimulation for bone formation. (F) Biocompatibility and antibacterial assay of the piezoelectric heterojunctions in vitro. (G) Osteogenic differentiation of piezoelectric heterojunctions in vitro. Reproduced with permission [205]. Copyright 2024, Wiley.

In essence, osseointegration is a complex healing process between bone and implants, encompassing a sequence of biological events such as an initial inflammatory response, angiogenesis, osteogenic differentiation, and osteogenesis [201]. To date, there have been a large number of studies focused on the surface and composite modification of Ti-based implants to facilitate osseointegration (Fig. 11A). Increasing evidence indicates that the physicochemical properties of implant surfaces, encompassing surface characteristics (e.g., charge and wettability), surface topography (e.g., micro/nanostructures and geometry), and surface chemical composition (e.g., bioactive ions such as calcium, phosphorus, silicon, strontium, magnesium, zinc, and copper; bioactive peptides such as BMP-2 and RGD), as well as their mechanical strength, are essential for successful osteointegration [2,104,201]. The great advances in the elucidation of osseointegration processes and the development of diverse modification strategies have inspired researchers to integrate osteogenesis promotion and PDT to endow implants with dual functions. For instance, by emulating the 3D fibrous architecture of the ECM in bone regeneration, Wu et al. [90] created a biomimetic micro/nano-TiO_2−*x*_ heterostructure coating that not only demonstrated photothermal/photodynamic synergistic antibacterial properties but also remarkably enhanced the proliferation and osteogenic differentiation of osteoblasts (Fig. 11B). In a separate investigation, the Ni(OH)₂@CaTiO₃ heterostructure constructed on a Ti implant efficiently segregated photoinduced electron-hole pairs, resulting in adequate generation of ROS, which facilitated efficient photoactivated bacterial inactivation. Concurrently, the calcium-rich, mildly alkaline surface offered by Ni(OH)₂@CaTiO₃ established an osteogenic microenvironment to enhance the adhesion, proliferation, and differentiation of MC3T3-E1 cells, as well as the up-regulation of osteogenesis-related gene expressions, thereby greatly expediting new bone formation and in vivo osseointegration of Ti implants [202]. Furthermore, photoelectron-converting materials that demonstrate precise spatiotemporal control over electrosignal-mediated osteogenesis have received attention. Upon light irradiation, photoinduced electron transfer forms a localized electric field around the implant. The generated photoelectrons can depolarize membrane potentials and activate voltage-gated calcium channels (VGCCs), thereby facilitating intracellular Ca^2+^ influx. This cascade activates the Wnt/Ca^2+^ signaling pathway, ultimately modulating downstream genes governing osteogenic differentiation, such as RUNX2 [4]. Leveraging the osteogenic effects of photoelectrons, the Au-RE/TiO_2_ implant could sequentially eradicate biofilms and regulate osteogenesis, providing new solutions to fabricate efficient dual-functional implant surfaces (Fig. 11C) [4].

Recently, inspired by the inherent piezoelectric properties of natural bone tissues, electrical stimulation has emerged as a frontier strategy for bone tissue engineering [203]. Piezoelectric materials convert mechanical loading into bioelectrical cues that promote osteogenic differentiation, thus accelerating bone regeneration [204]. Piezoelectric heterojunction implants featuring photo-mechano-electrical coupling capabilities offer dual functionality: promoting osteogenesis while eradicating pathogenic bacteria, representing a promising solution to the complex challenge of repairing infected bone defects [205]. Bi_2_WO_6_ is a narrow-bandgap semiconductor material with piezoelectric properties and good biocompatibility. In the pioneering work of Fan et al. [205], piezoelectric TiO_2_/Bi_2_WO_6_ heterojunctions were fabricated on Ti implants via in situ hydrothermal growth of Bi_2_WO_6_ nanocrystals on TiO_2_ nanowire-functionalized surfaces. The ethylene glycol treatment during heating reduced the oxygen ions in metallic oxides into oxygen, leading to the formation of oxygen vacancies, which endowed the heterojunctions with improved ability to absorb NIR light and produce heat. Concurrently, the interfacial built-in electric fields within the heterojunctions facilitated charge transfer, effectively promoting the separation of photogenerated carriers and ROS generation. Therefore, with the combined bactericidal effects of PTT and PDT upon NIR light irradiation, the heterojunctions demonstrated high promise for bacterial eradication in recurrent implant infections. Furthermore, harnessing Bi_2_WO_6_’s excellent piezoelectric property, the heterojunctions converted the mechanical forces exerted by cell growth on their surfaces into electrical signals, achieving spatiotemporal modulation of BMSC osteogenic fates through the activation of the calcium enrichment pathway and PI3K-AKT pathway, thereby promoting bone regeneration and osseointegration (Fig. 11D to F).

Beyond the direct role of osteoblasts in osseointegration, the osteoimmune microenvironment profoundly influences implant-associated immune responses and bone formation [206]. As pivotal effector cells in immune response, macrophages exhibit a dynamic balance and can polarize into inflammatory (M1) or anti-inflammatory (M2) phenotypes under various stimuli. M2 macrophages orchestrate inflammation resolution and osteogenic activation through coordinated release of anti-inflammatory (e.g., IL-10) and pro-osteogenic [e.g., vascular endothelial growth factor (VEGF) and BMP-2] cytokines, establishing a favorable osteoimmune environment for bone regeneration [207]. Consequently, efficient and timely conversion of M1 to M2 macrophages is crucial for peri-implant tissue healing and osseointegration [208]. Previous discussions have documented PDT’s capacity to enhance antibacterial efficacy through M1 macrophage activation. However, balancing the proinflammatory response (predominantly M1) required for bacterial clearance with the specific immune microenvironment (predominantly M2) essential for successful osseointegration remains a thorny challenge. ROS activate the nuclear factor-κB (NF-κB) pathway, triggering proinflammatory cytokine release and M1 polarization, thereby driving inflammatory responses. Scavenging excess ROS has proven effective in mitigating inflammation and preventing tissue damage [209]. Thus, precise ROS modulation may enable optimal transition between these immune states. Fortunately, advances in material science have yielded rationally engineered 2D nanozymes exhibiting exceptional bidirectional redox regulation. These agents not only possess excellent enzyme-mimetic activities, including POD, CAT, and SOD for ROS clearance, but also exhibit ROS-generating capacity under exogenous photodynamic stimulation, enabling efficient pathogen eradication [210]. For instance, Wang et al. [211] developed selenium-doped CeO₂ NPs where selenium incorporation modulated band structure to broaden absorption spectra, permitting efficient NIR-activatable PDT. Within this ROS-balancing system, CeO₂ NPs demonstrated dual antagonistic functions: passive ROS production and self-quenching. As nanozymes, they exhibited CAT and SOD activities that rapidly eliminated wound-surface ROS while generating O₂. Upon illumination, their roles switched and functioned as photosensitizers, producing ^1^O₂ using O₂ as substrate. Following NIR irradiation, CeO₂ NPs promptly scavenged ROS to alleviate oxidative stress-induced cellular damage and accelerated macrophage polarization from M1 to M2 phenotypes, thereby facilitating tissue healing. This innovative work exemplifies how 2 seemingly opposing mechanisms, “PDT and ROS scavenging”, work in synergy to promote healing by finely tuning the balance between the generation and removal of both endogenous and exogenous ROS, which provides valuable insights for developing Ti implants that simultaneously prevent infections and promote bone healing.

In addition to implant material design, light-mediated enhancement of osseointegration merits sufficient consideration. Substantial evidence confirms that LLLT promotes bone healing and osseointegration through photobiomodulation [212]. This process involves 3 fundamental events: photon absorption by cellular chromophores, photophysical activation of signaling pathways, and downstream regulation of cellular responses [213]. Mechanistically, LLLT enhances cell proliferation, ATP synthesis, mitochondrial activity, and osteoblast differentiation in LLLT-treated cells via elevated intracellular ROS-mediated signaling [214]. Bai et al. [215] demonstrated that LLLT accelerates vascularized bone regeneration through coupled angiogenesis–osteogenesis, identifying the ROS/HIF-1α pathway as essential for these effects. Li et al. [216] further established that 650-nm photobiomodulation treatment (6 J/cm^2^) potentiates osteogenic differentiation in osteoporotic BMSCs, suggesting autophagy activation as a contributory mechanism. Despite these advances, the precise mechanistic basis of LLLT-mediated benefits in osteogenesis and osseointegration remains incompletely characterized. Although parameters of 635- to 980-nm wavelength, 40- to 100-mW power, and <100 J/cm^2^ energy density are commonly employed, considerable therapeutic outcome heterogeneity persists due to the variations of LLLT protocols [213]. Collectively, LLLT represents a promising strategy for accelerating osseointegration. The integration of PDT with LLLT offers particular potential for dual-functional implants, as demonstrated by He et al. [79]: A Ce6-loaded GelMA hydrogel coating on Ti implants achieved both efficient antibacterial efficacy (PDT: 660 nm, 1 W/cm^2^) and satisfactory tissue healing (LLLT: 660 nm, 100 mW/cm^2^) through adjustable laser parameters. Future research should prioritize incorporating LLLT into standardized implant surgical protocols to optimize clinical osseointegration outcomes.

## Summary and Future Perspectives

Due to its broad-spectrum antimicrobial effectiveness, noninvasive nature, and minimal propensity to foster drug resistance, PDT has increasingly attracted substantial interest in infection control, showcasing enormous therapeutic potential. The utilization of PDT on Ti implants to combat bacterial infections and facilitate optimal circumstances for eventual osseointegration is a highly promising therapeutic approach. This review initially delineates the primary mechanisms of PDT for bacterial elimination, subsequently providing an exhaustive examination of prevalent organic and inorganic photosensitizers utilized in Ti implants. While PDT has demonstrated remarkable advantages for the prevention of Ti osteoimplant infections, it faces certain limitations. The effectiveness of PDT is directly correlated with ROS yields. However, conventional organic photosensitizers frequently suffer from ACQ, which markedly compromises their photodynamic efficiency. The photophysical mechanisms suggest that a high quantum efficiency for ISC and a long-lasting triplet state are expected to amplify ROS production. AIE-active photosensitizers with exceptional photostability and augmented ROS generation through aggregation-enhanced ISC therefore represent a promising alternative [171]. Furthermore, their excellent fluorescence emission capacities enable them as powerful fluorescent tools for microbial detection. The incorporation of AIE-active photosensitizers onto implant surfaces may establish a reliable theranostic platform able to rapidly detect biofilm infections, permitting precise phototherapy timing, thereby circumventing the delayed intervention inherent in traditional symptom-based treatment. Metal-based photosensitizers offer versatile options for PDT on Ti implants. However, their design must reconcile the inherent biocompatibility challenges arising from slow physiological degradation with the cytotoxicity caused by rapid metal ion leaching and excessive accumulation. Osseointegration requires a weakly alkaline microenvironment, while bacterial infections typically induce acidosis that hinders mineral deposition and bone regeneration. Consequently, alkaline metal-based photosensitizers warrant focused investigation to neutralize pathological acidity and create an optimal bone regeneration microenvironment. Furthermore, the remarkable bioactivity of physiological metal ions (Ca^2+^, Mg^2+^, Zn^2+^, Cu^2+^) warrants strategic incorporation into photosensitizer design to potentiate osteogenesis, immunomodulation, and angiogenic processes.

Oxygen availability is universally acknowledged as a critical limiting factor in ROS generation. Pathogenic bacteria readily adhere to Ti implant interfaces, inducing biofilm formation that severely exacerbates hypoxic microenvironments. To mitigate the hypoxic conditions induced by biofilms, 2 principal strategies have been devised: augmenting oxygen delivery and facilitating in situ oxygen generation. Although these approaches have demonstrated excellent efficacy in antitumor therapy, their implementation in implant-associated infections is still insufficiently explored. Meanwhile, it is necessary to explore the effects of hyperoxic conditions on osteoblast activity and angiogenesis, which are critical for osseointegration. Developing effective surface engineering techniques is crucial for achieving stable immobilization of oxygen delivery vehicles or oxygenating agents onto biomedical Ti surfaces. Various techniques, including physical coating, surface grafting, and covalent bonding, have been extensively employed to facilitate the effective loading or anchoring of photosensitizers onto bioinert Ti surfaces. The advancement and utilization of biocompatible nanocarriers, such as PDA and MSNs, offer viable solutions to the intrinsic constraints of photosensitizers, including low aqueous solubility, insufficient stability, and inadequate biocompatibility. Future research should focus on developing intelligent nanocarriers that enable microenvironment-responsive drug release and bacterial targeting, thereby enhancing the therapeutic efficacy of PDT.

Appropriate illumination parameters are a critical determinant for effective and safe PDT. Current studies exhibit considerable variability in the laser configuration, such as wavelength, power density, exposure duration, and beam uniformity of light sources, even when utilizing the identical photosensitizers, which constrains their applicability as reference benchmarks for subsequent research. Augmenting light intensity or extending illumination duration enhances antimicrobial efficacy; however, it inevitably exacerbates collateral damage to surrounding healthy tissues. The absence of standardized criteria for evaluating such damage underscores the need for further study to establish well-defined safety thresholds. To overcome the limited penetration depth of conventional excitation wavelengths, recent strategies emphasize the development of photosensitizers activated by NIR light, illustrated by various modifications of TiO_2_ and heterojunction engineering. NIR-responsive UCNPs, serving as wavelength converters in PDT systems, have recently gained interest due to their excellent ability to enhance NIR light utilization by photosensitizers. Innovations in x-ray-activated PDT utilize the extensive tissue penetration and imaging capabilities of x-rays, showing great potential to transform PDT applications for Ti-based implants. Future development of x-ray-excitable photosensitizers must meticulously consider biosafety concerns, particularly the carcinogenic dangers associated with repeated x-ray exposure.

The combination of PDT with other therapeutic modalities has been extensively explored to improve antibacterial efficacy. By leveraging the benefits of individual therapy and addressing its deficiencies, synergistic effects yield mutual benefits, drastically enhancing antibacterial treatment. The PDT/PTT strategy presents the most promising potential due to a comparable excitation window in the NIR spectrum. Notably, certain materials intrinsically function as dual photosensitizing and photothermal agents, exemplified by ICG, metal-based photosensitizers, and carbon-based nanomaterials. These merit prioritization in future research endeavors. Gaseous compounds such as NO, CO, and H_2_S can enhance the bactericidal efficacy of ROS and play a regulatory role as a signaling molecule at their physiological concentration. Therefore, the development of an intelligent gas-releasing system is anticipated to align with the elimination of early-stage implant infections and the ensuing bone integration phase. Noteworthily, the immune responses induced by PDT are receiving increasing interest in both antitumor and anti-infective applications. PDT combined with immunotherapy has exhibited huge success in combating cancers and inducing immunological memory, hence ensuring prolonged treatment effectiveness. However, PDT-mediated immunotherapy has not been comprehensively elucidated regarding its efficacy against biofilm infections, necessitating further work to validate its potential for preventing infection occurrence and recurrence. Given the indispensable effects of proinflammatory immune microenvironments for infection removal and anti-inflammatory immune microenvironments for osseointegration, future research should prioritize smart PDT-enabled Ti implants capable of dynamically balancing these dual immune phases to facilitate seamless transition from anti-infection to bone regeneration. Notably, nanozymes with concurrent enzyme-mimetic and photosensitizing activities that enable efficient ROS production upon light activation while timely scavenging excess ROS without light warrant prioritized investigation as core functional components for precise immune modulation.

To date, the development of photodynamic antibacterial therapy remains in its nascent stage. Despite the promising results of the aforementioned studies, a paucity of compelling clinical evidence requires additional research before PDT may be integrated into clinical protocols for the management of implant-associated infections. To facilitate the clinical translation of PDT in preventing Ti osteoimplant infections, it is imperative to conduct standardized comparative studies that systematically evaluate the critical physicochemical parameters and antibacterial efficacies of photosensitizers, along with the precise doses, power densities, frequencies, and durations of irradiation concerning the illumination protocol. These investigations are critical for determining suitable photosensitizers for clinical application and establishing the optimal illumination parameters for distinct photosensitizers. Most recent studies are primarily focused on achieving optimal antibacterial efficacy. Nevertheless, from an engineering perspective, developing facile and cost-effective methods to integrate PDT platforms onto implant surfaces while ensuring their efficacy and long-term stability is crucial for the widespread adoption and clinical translation of PDT. This should also be a key emphasis for future research. Most importantly, inadequate biological examinations in existing research necessitate focused attention on the long-term study of toxicology. Apart from evaluating PDT’s adverse effects on local tissues at the implantation site, a comprehensive toxicological assessment of photosensitizers and their nanocarriers is imperative, especially concerning their metabolic pathways and biodistribution characteristics. Future research should systematically elucidate the degradation processes and pharmacokinetics of these compounds. For the ultimate goal of stable osseointegration, dual-functional Ti implants with integrated antibacterial and osseointegration capabilities ought to be a burgeoning trend in clinical implant design. With the rapid progress in nanomedicine and surface engineering technology, PDT is expected to witness expanded therapeutic applications in orthopedic and dental implantology.
